# Selective depletion of *Campylobacter jejuni* via T6SS dependent functionality: an approach for improving chickens gut health

**DOI:** 10.1186/s13099-024-00628-6

**Published:** 2024-07-12

**Authors:** Subhadeep Gupta, Prakash Biswas, Bishnu Das, Samiran Mondal, Parna Gupta, Dipjyoti Das, Amirul Islam Mallick

**Affiliations:** 1 Department of Biological Sciences, Indian Institute of Science Education and Research Kolkata, Mohanpur, Nadia, West Bengal 741246 India; 2 Department of Chemical Sciences, Indian Institute of Science Education and Research Kolkata, Mohanpur, Nadia, West Bengal 741246 India; 3 Department of Veterinary Pathology, West Bengal University of Animal and Fishery Sciences, Kolkata, West Bengal 700037 India

**Keywords:** Microbial competition, Type-VI Secretion System, Antibiotic alternative, Chickens gut health

## Abstract

The targeted depletion of potential gut pathogens is often challenging because of their intrinsic ability to thrive in harsh gut environments. Earlier, we showed that *Campylobacter jejuni* (*C. jejuni*) exclusively uses the Type-VI Secretion System (T6SS) to target its prey such as *Escherichia coli (E. coli)*, and phenotypic differences between T6SS-negative and T6SS-positive *C. jejuni* isolates toward bile salt sensitivity. However, it remains unclear how the target-driven T6SS functionality prevails in a polymicrobial gut environment. Here, we investigated the fate of microbial competition in an altered gut environment via bacterial T6SS using a T6SS-negative and -positive *C. jejuni* or its isogenic mutant of the hemolysin-coregulated protein (*hcp*). We showed that in the presence of bile salt and prey bacteria (*E. coli*), T6SS-positive *C. jejuni* experiences enhanced intracellular stress leading to cell death. Intracellular tracking of fluorophore-conjugated bile salts confirmed that T6SS-mediated bile salt influx into *C. jejuni* can enhance intracellular oxidative stress, affecting *C. jejuni* viability. We further investigated whether the T6SS activity in the presence of prey (*E. coli*) perturb*s* the in vivo colonization of *C. jejuni*. Using chickens as primary hosts of *C. jejuni* and non-pathogenic *E. coli* as prey, we showed a marked reduction of *C. jejuni* load in chickens cecum when bile salt solution was administered orally. Analysis of local antibody responses and pro-inflammatory gene expression showed a reduced risk of tissue damage, indicating that T6SS activity in the complex gut environment can be exploited as a possible measure to clear the persistent colonization of *C. jejuni* in chickens.

**Supplementary Information:**

The online version contains supplementary material available at 10.1186/s13099-024-00628-6.

## Highlights

1. Differential response of *C. jejuni*T6SS under bile salt stress.

2. T6SS function was probed by deleting its essential functional subunit, Hcp.

3. The presence of prey bacteria facilitates enhanced bile salt influx and oxidative stress in T6SS-positive *C. jejuni.*

4. Decreased colonization of *C. jejuni*in chickens by tuning T6SS activity.

**Supplementary Information:**

The online version contains supplementary material available at 10.1186/s13099-024-00628-6.

## Background

Commensal bacteria within the gut engage in a symbiotic interaction with the host, significantly influencing gut health and overall fitness [1, 2]. These effects manifest through the modulation of various host functions, such as development, metabolism, and immunity [3, 4]. Conversely, common gut pathogens possess various inherent mechanisms to circumvent host defenses, enhance host pathogenicity, and withstand exposure to diverse environmental stressors [5]. Pathogenic gut bacteria often use committed secretion systems for their survival, disease progression, and competitive advantages [6, 7]. The bacterial Type VI Secretion System (T6SS) is noted for its functional versatility such as self-survival and secretion of effector molecules towards competitive fitness advantages in a complex gut microenvironment [8, 9]. The bacterial T6SS functionality via dynamic cycles of assembly, contraction, and disassembly targets both prokaryotic, eukaryotic and even ‘sister’ populations within the same ecological niche [10–16]. However, recent studies suggest that the T6SS functionality of a range of gut pathogens including *C. jejuni* can be influenced by different stress variables, such as oxidative and chemical stressors, heat-shock factors, osmotic and low-pH stress [17–23].

As key oxidative stressors naturally present in the gut, bile salts share a complex relationship with resident gut-microflora. In particular, bacterial metabolism and their relative abundance shape the spatial composition of bile salts (or vis-à-vis) in the gastrointestinal tract to maintain gut homeostasis [24]. Specifically, gut microbes utilize several mechanisms to adopt bile salt resistance, which is considered an important selective pressure in modulating the gut microbiome population [25]. The differential ability of bacteria to withstand bile salt resistance is mediated by activating efflux pumps [26, 27], alteration of membrane protein [28, 29], lipid composition [30–33] and cell wall composition [34]. Previously, we found phenotypic differences between T6SS-negative and T6SS-positive *C. jejuni* isolates toward bile salt sensitivity. Notably, in the presence of a target (prey), T6SS-positive *C. jejuni* was less tolerant toward bile salts than in the case without prey [35]. However, it remains unclear whether the T6SS functionality could affect *C. jejuni* survivability under a complex and polymicrobial gut environment in an in vivo conditions. Another question is whether bile salt tolerance relies on the direct or indirect involvement of target-driven T6SS functionality.

To this end, we used non-pathogenic *Escherichia coli* (*E. coli*-DH5α) as prey for the T6SS-positive reference strain of *C. jejuni* (ATCC 43,431-TGH 9011) and another reference strain of *C. jejuni* (NCTC 11,168; GenBank ID: AL111168.1), which lacks the complete T6SS sequence as a negative control to assess stress tolerance using chickens as an in vivo model host. Given that the hemolysin-coregulated protein (Hcp) of *C. jejuni* is the key functional protein of the T6SS system [36, 37], we also confirmed the overall functionality of T6SS in an in vitro setup using an *hcp* deletion mutant (Δ*hcp*) of the isogenic background of T6SS-positive *C. jejuni* (ATCC 43431-TGH 9011).

The data generated from in vitro competition of *C. jejuni* and *E. coli*, and enhanced intracellular accumulation of iridium-conjugated bile salt with high-level production of reactive oxygen species (ROS) suggest that T6SS functionality could entail “a cost” for T6SS-positive *C. jejuni* cells when bile salts are present. Finally, in vivo chickens experiment demonstrated that the birds that received T6SS-positive *C. jejuni* along with *E. coli*, showed ~ 1.0 log reduction in the T6SS-positive population with a low level of local antibody responses (sIgA) and pro-inflammatory cytokine gene expression. Together with efficient clearance of *C. jejuni*, low-level sIgA responses and pro-inflammatory gene expression, suggest that T6SS functionality could restore chickens gut health and potentially reduce the risk of food-borne transmission of *C. jejuni* to humans.

In summary, we propose that utilizing the prey-driven T6SS functionality of *C. jejuni* can help us develop new dietary formulations that protect birds from a range of other T6SS-harboring enteric pathogens. Although the acquisition of bile tolerance is a natural process by major gut microbiota, bile salt adaptation may affect the probiotic traits of useful gut microbes [38]; therefore, further investigation is needed to determine the appropriate form and optimized dose for the exogenous application of bile salt.

## Methods

### Bacterial strains and plasmids

#### Bacteria

*C. jejuni* reference strain (TGH9011) was obtained from BEI Resources, NIAID, NIH: *C. jejuni* subsp. *jejuni*, Strain TGH 9011, NR-4082, and the T6SS-negative *C. jejuni* strain (NCTC 11168; GenBank ID: AL111168.1) was obtained from the NIH Biodefense and Emerging Infections Research Repository, NIAID, NIH: *C. jejuni* subsp. *jejuni*, Strain NCTC 11,168, NR-126. The *E. coli* (DH5α) cells were procured from BioBharati Life Science, India.

#### Bacterial vectors

The *pTurbo*GFP-B plasmid was transformed into *E. coli* (DH5α) cells to generate GFP-expressing *E. coli* (r*E. coli*). The *pTurbo*GFP-B was kindly provided by Dr. Partha Pratim Datta, IISER Kolkata. Details of the bacterial transformation procedure can be found in our previous work [35, 39]. The *p*JMK30 plasmid was used as the source of the kanamycin gene cassette to generate an isogenic mutant of *C. jejuni* (kindly provided by Prof. Andrey Karlyshev, Kingston University, London, UK).

#### Cell lines

Chicken Embryonic Intestinal Cells (CEICs) were harvested from 19-day-old chicken embryos. Cells were grown in complete RPMI with 10% FBS (v/v), 100 IU/mL of penicillin, and 100 µg/mL of streptomycin at 37 °C with 5% CO_2_ supplementation [40].

#### Generation of hcp mutant C. jejuni(Δ*hcp*)

Targeted gene deletion of *hcp* was performed by homologus recombination methods using a kanamycin-resistant (*Km*^R^) cassette from *p*JMK30 vector [41]. The *Km*^R^ cassette was amplified using primers containing the EcoRI restriction sites. The upstream and downstream primers (with RE sites, matched to *Km*^*R*^ cassette) were used to amplify the flanking regions of the *hcp* gene. Further, the *p*BSK-II(+) vector was used to ligate the PCR-amplified fragments before transformation into *E. coli* (Top10). Next, a purified plasmid from *E. coli* was used to transform into *C. jejuni* (TGH 43431) via electroporation. Briefly, *C. jejuni* (TGH 43431) culture was resuspended in buffer (mixture of 15% glycerol and 272 mM sucrose), followed by three times washing with the same buffer, and then a 50 µL aliquot was used for each transformation. The mixture was shifted to a chilled electroporation cuvette following the addition of ligated plasmid (0.5 µg in 5 µL). After electroporation, fresh Brucella broth (100 µL) was introduced into the cuvette, followed by plating the bacterial suspension on a blood agar plate, which was further followed by incubation at 37 °C under microaerobic conditions. After visible bacterial growth on the Blood Agar plate, the bacteria were spread onto a kanamycin-supplemented blood agar plate and cultured for 5–6 days under microaerobic conditions at 37 °C. The confirmation of correct gene orientation was done by Sanger sequencing and PCR, followed by transcriptome analysis of the target gene (*hcp*) and western blot analysis of the whole cell lysate of the mutant strain. Primers are listed in Supplementary table S3.

#### Chemical and reagents

All analytical grade chemicals and reagents were used in this study, obtained from commercial suppliers, and used without further purification unless otherwise mentioned. The chemicals were procured from Sigma-Aldrich (4′-Chloro-2, 2′:6′2′′-terpyridine), Arora-Matthey Limited (IrCl_3_.3H_2_O), and Merck (KOH), and were used without further modification. The solvents were obtained from Sigma-Aldrich (d_6_-DMSO, CDCl_3_), SD Fine-Chem Limited (dichloromethane, methanol, and acetone), and used after passing through hot water Na_2_SO_4_. Bile salt mixture (1:1 mixture of Sodium Deoxycholate and Sodium Cholate) was procured from Himedia, India.

### In vitro assessment of *C. jejuni* response to bile salt stress

#### Assessing the changes in bacterial morphology

Bile salt-induced changes in *C. jejuni* morphology were visualized by Field Emission Scanning Electron Microscopy (FESEM). Samples were prepared according to a method published elsewhere [39]. In brief, equal number of *C. jejuni* (1 × 10^7^ CFU/mL), either T6SS-positive or T6SS-negative or Δ*hcp* mutant of *C. jejuni* was co-incubated with *E. coli* (2 × 10^8^ CFU/mL) in Mueller-Hinton (MH) broth (HiMedia, India) supplemented with 0.1% (w/v) of bile salt mixture (Grade III, HiMedia). After 7 h of co-incubation under microaerobic conditions, bile salt-treated *C. jejuni* cells were fixed using 2.5% (v/v) glutaraldehyde, followed by three times washing using PBS. Next, fixed bacterial cells were incubated serially in 35%, 50%, 70%, and 95% ethanol for 10 min each, followed by 1 h incubation with 100% ethanol for complete dehydration. Eventually, the dehydrated cells were vacuum-dried for 1 h, followed by the fixation on aluminum stubs with silver conductive paint, to perform sputter-coating with platinum. Further, microscopy was performed using a Supra 55 Carl Zeiss scanning electron microscope (Carl Zeiss, Germany).

#### Visualization and quantification of intracellular reactive oxygen species (ROS) by fluorescence imaging

The samples collected from each group were prepared according to a method published elsewhere [39]. In brief, following the coculture of *C. jejuni* (1 × 10^7^ CFU/mL) and *E. coli* (2 × 10^8^ CFU/mL) for 7 h at 37 ^o^C (at 10% CO_2_, 5% O_2_, and 85% N_2_), 100 µL from the mixture was plated onto bile salt added (0.1%; w/v) MH agar and grown for ~ 48 h. Further, ~ 10 individual, *C. jejuni* colonies were picked up from the same plates based on green-white screening (*C. jejuni*: white colonies vs. r*E.coli*: green colonies) and dissolved in 1 mL of MH broth. A total of 6 plates were processed under similar conditions (*n* = 6) and the absorbance of each colony dissolved in MH broth was normalized to ~ 0.5 OD_600_. A total of 500 µL from each sample was centrifuged and next the pellet was incubated with 200 µL of 2′, 7′-dichlorodihydrofluorescein diacetate (H_2_DCFDA; 1 µM) and incubated for 1 h. Following washing, 4% PFA was used to fix the cells. Next, 4 µL of the fixed, stained cells were affixed to a glass slide employing Vecta-shield mounting medium (Vector Laboratories, USA) for fluorescence imaging under Axio observer equipped with ApoTome module (Carl Zeiss, Germany) in FITC filter and 100 µL of cell pellet was subjected to fluorescence spectrophotometry using the Spectramax M2e Multi Detection Microplate Readers (USA) (at λex: 485 nm; λem: 535 nm).

#### Effect of bile salt-induced stress on C. jejuni growth profile

To examine the effects of bile salt-induced stress, T6SS-positive (TGH 9011), Δ*hcp* (TGH 9011), and T6SS-negative (NCTC 11,168) strains of *C. jejuni* were used. An equal number of *C. jejuni* (1 × 10^7^ CFU/mL), either T6SS-positive or T6SS-negative or Δ*hcp* mutant of *C. jejuni* was co-incubated with *E. coli* (2 × 10^8^ CFU/mL) were cocultured in MH broth (HiMedia, India) supplemented with different concentrations of bile salt ranging from 0.05% to −0.125% (w/v). After 7 h of co-incubation under microaerobic conditions, followed by 1.0 mL from each tube was diluted serially and 50 µL from the last dilution was plated onto MH agar plate. The plates were incubated for ~ 48 h at microaerobic conditions and the colony was counted based on the green-white screening. Further, the colonies on the plates were enumerated individually and plotted as the average CFU/mL with standard deviation (± SD) .

### In vitro tracking and visualization of bile salt transport to *C. jejuni*

#### Synthesis of iridium conjugated bile salt (Ir-TBS) complex

To prepare TBS, bile salt solution (500 mg, 1.1620 mmol) was initially introduced into a stirred mixture of powdered KOH (391.1 mg, 6.972 mmol) in DMSO (20 mL) at 60 °C. Following a 20-minute interval, 4’-chloro-2,2’:6’,2″-terpyridine (777.7 mg, 2.9051 mmol) was introduced into the mixture and stirred for 3 days at 60 °C. Subsequently, the reaction mixture was poured into 100 mL of deionized water. Adjustment of the solution’s pH to 7 was achieved using concentrated HCl, followed by filtration to remove the aqueous layer. Further, the compound underwent a washing process with deionized water, followed by vacuum drying and purification through column chromatography, employing a DCM: Hexane mixture (95:5) as the eluting solvent. TBS (50 mg, 0.0444 mmol) was subjected to reflux at 80 °C for 4 h with [Ir(ppy)_2_]_2_Cl_2_ (71.5 mg, 0.1334 mmol) in a solution of dichloromethane and methanol (3:1). After removing the solvent at low pressure the compound underwent purification through preparative thin-layer chromatography, utilizing a dichloromethane mixture containing 7% methanol. The final compound was obtained as the second major fraction [42–46].

#### Biophysical characterization of Ir-TBS complex

The presence and integrity of the Ir-TBS complex were validated using Electrospray ionization - Mass Spectrometer (ESI-MS) (positive mode electrospray ionization with the Bruker maXis IITM instrument, USA) and Nuclear Magnetic Resonance (NMR) spectroscopy. The NMR spectra (^1^H and ^13^C) were acquired on JEOL ECS 400 (Japan) and Bruker-500 spectrometers (Bruker, USA), respectively. The Perkin Elmer (USA) instrument was used to obtain the Fourier Transform Infrared (FT-IR) spectra. The Jasco V 670 spectrophotometer (Japan) was used to measure UV-Vis spectra of both TBS and Ir-TBS (1 × 10^− 5^ M). The fluorescence spectra of TBS and Ir-TBS (1 × 10^− 5^ M) in solution were recorded using a Fluoromax spectrofluorometer (Horiba Jobin Yvon, Japan). The stability of Ir-TBS was assessed over four days using time-dependent ^1^H NMR and UV-vis spectroscopy.

#### In vitro tracking of bile salt influx into *C. jejuni*

To track and visualize intracellular transport of bile salts, *C. jejuni* and *E. coli* were cocultured in MH broth supplemented with Ir-TBS (0.05%, w/v) and kept at 37 °C. To check the Ir-TBS influx, bacterial cultures were collected and washed, and the fluorescence signal of Ir-TBS complex inside the bacteria was measured by scanning the fluorescence spectra (λ_ex_:400 nm; λ_em_:580 nm). Further, to visualize the intracellular accumulation of Ir-TBS, *C. jejuni* cells were harvested 6 h post-treatment and processed for Confocal Laser Scanning Microscopy (CLSM), as described earlier. After fixation, the bacterial cells were examined under a Leica confocal microscope (Germany) (λ_ex_:405 nm; λ_em_:580 nm), and images were captured.

#### Assessing the effect of T6SS-mediated predation on *C. jejuni *invasion to primary CEICs

The number of *C. jejuni* associated with CEICs (adhered and invaded) was determined according to the protocol published elsewhere [40]. In brief, a confluent monolayer of primary CEICs was infected with *C. jejuni* with or without the presence of *E. coli* at MOI 300:1 for 7 h at 37 °C and 5% CO_2_ in RPMI supplemented with 10% FBS and 0.05% bile salt. Next, the medium was discarded, followed by washing with 1X PBS. The cells were lysed using 1% Triton X-100 to release intracellular bacteria, enhancing the release into the pool of adhered bacteria. The mixture was then consecutively diluted (10-fold) and plated on MH agar plate. The *C. jejuni* colonies on the plate were enumerated and reported as the average CFU/mL ± SD. To determine the number of intracellular bacteria, we also performed gentamicin protection assay [40]. For this, after 7 h of infection, the cells were incubated for 2 h with gentamicin (150 µg/mL) to kill adhered bacteria. Further, cells were washed, followed by lysing using Triton X-100 as previously described.

#### Imaging method

Monolayers of primary CEIC cells were grown on coverslips at the density of 1.2 × 10^6^ cells/well. Prior to infection, *C. jejuni* cells were labeled with 4′,6-diamidino-2-phenylindole (DAPI; 5 µg/mL). Further, primary CEICs were treated with various sets of treatment groups: *C. jejuni* only, *C. jejuni* with bile salt (0.05%, w/v), *C. jejuni* with *E. coli*, *C. jejuni* with *E. coli* and bile salt (0.05%, w/v) for 7 h under 5% CO_2_ pressure. After removing the medium, cells underwent a PBS wash before fixation with 4% PFA for 20 min. Subsequent to fixation, phalloidin 647 (Abcam, UK) staining was conducted for 1 h, followed by another wash, and finally, mounting on slides using a mounting medium (Vectashield). Images were captured using a Leica confocal microscope under a DAPI filter (λ_ex_:358 nm and λ_em_:461 nm for *C. jejuni*) and a phalloidin filter (λ_ex_: 647 nm, λ_em_: 668 nm for CEICs) and further processed using Fiji software.

### In vivo assessment of T6SS functionality in selective killing of *C. jejuni *in chickens

#### Experimental birds and housing conditions

For this present study, we used 9 birds per experimental group (a total of 8 groups). The chickens experiments were conducted separately with both T6SS-positive (4 groups) and T6SS-negative (4 groups) under similar experimental conditions. Thus, the total number of birds used = (9 birds per group × 4 × 2 = 72). Throughout the trial, the birds were housed in a deep litter system and provided with unrestricted access to an antibiotic-free mash diet. The specific feed composition used for this study is detailed in Table S2.

#### Experimental groups

After seven days of acclimatization, the birds were divided into four different groups (Groups A, B, C, and D). From Day 7 to Day 14, Group C and Group D birds were administered with bile salt dissolved in PBS (0.2%, w/v) daily, while Group A and Group B received only PBS. Furthermore, from day 15 to day 35, birds from different groups received the following treatments consecutively for three days per week. Group A: *C. jejuni*; Group B: Mixture of *C. jejuni* and *E. coli;* Group C: Mixture of *C. jejuni* and bile salt; Group D: Mixture of *C. jejuni, E. coli*, and bile salt.

#### Feeding regimens

Chicks from different groups were orally administered the following treatments. Group A: 1 × 10^7^ CFU *C. jejuni* in PBS; Group B: 1 × 10^7^ CFU *C. jejuni +* 2 × 10^8^ CFU *E. coli* in PBS; Group C: 1 × 10^7^ CFU *C. jejuni* + 0.2% (w/v) bile salt mixture in PBS; Group D: 1 × 10^7^ CFU *C. jejuni +* 2 × 10^8^ CFU *E. coli* + 0.2% (w/v) bile salt mixture in PBS.

#### Sample collection (cecal content, gastric lavages, and fecal materials)

On day 30 after feeding, fresh fecal samples were obtained from each bird in every group at two distinct time intervals (3 h and 4 h). On day 35, all birds were euthanized, 3 h after the last feeding. After sacrifice, ~ 5 g of cecum tissue sample was obtained from each bird and kept at -20 ºC in RNA-later™ (Qiagen, Germany). The cecal tissue samples for histopathological examination were acquired and 10% neutral formalin buffer (NFB) was used for fixation for 48 h. Approximately 500 mg of cecal content was diluted in MH broth to measure *C. jejuni* load in the cecum. The intestinal lavages were obtained using a protocol established in our laboratory and were subsequently stored at -20 °C [47].

#### Assessing local antibody responses (sIgA) against C. jejuni in gastric lavages

To determine anti-*C. jejuni* sIgA antibody level in the intestine, lavage samples obtained from chickens in various experimental groups to perform an indirect ELISA [47]. In brief, maxisorp ELISA plates were coated with *C. jejuni* whole-cell lysate overnight at 4 °C prepared in carbonate-bicarbonate coating buffer (pH ~ 9.6). Next day, the plates were washed with PBS-Tween (PBS-T), followed by 1 h blocking at 37 °C with 5% Bovine Serum Albumin (BSA). After thorough washing of the wells, serially diluted intestinal lavage samples were added and allowed for 2 h incubation at room temperature (RT). After washing, the plates were probed with HRP-conjugated goat anti-chicken IgA secondary antibody (dilution: 1:2500; Thermo Fisher Scientific) for 1 h at RT. Subsequently, following three washes, TMB substrate was applied to each well, and the reaction was terminated by the addition of 50 µL of a stop solution comprising 1 M H_2_SO_4_. The absorbance was then determined utilizing a microplate reader (at 450 nm) from BioTek.

#### Determining the C. jejuni load

To enumerate *C. jejuni* load, fecal and cecal samples of orally administered birds in every group were collected on days 30 and 35, respectively. Approximately 500 mg of feces and cecal content were taken and dissolved in 1 mL of MH broth, followed by serial dilution, and plated onto blood-free Campylobacter selective media. Then, the plates were kept for incubation at 37 °C under microaerobic conditions, and colonies were counted after 48 h of incubation [48].

#### Determination of cytokine gene expression profile in cecal tissue by RT-qPCR

Total RNA was extracted from the cecal tissues using TRIzol (Invitrogen, USA). Reverse transcription of RNA for each sample was performed using a first-strand cDNA synthesis kit (BioBharati Life Science, India). To evaluate cytokine gene expression, RT-qPCR was performed using a primer set for chicken IL-8, IL-17 A, IL-6, and IL-1β, while the chicken β-actin gene was used as the internal control. The primers details used are listed in Table S3.

#### Histopathological analysis of cecal tissue

To perform histopathological examination, the cecal tissue was collected from the experimental birds on day 35 and fixed in 10% NFB for 48 h. After fixation, the tissue samples were washed overnight with slow-running tap water and subsequently dehydrated by passing them through a series of acetone grades (70–100%). Thereafter, The dried samples were placed in 100% benzene for 1 h to make them clear and transparent. Next, the samples were impregnated with melted paraffin (temperature 56 °C) and embedded in paraffin using metal molds to make a paraffin block. Paraffin-embedded samples were cut into thin slices (5 µM) using a rotary microtome and further processed for the standard de-paraffinization by immersion in xylene for 2 min. The deparaffinized tissue slices were then hydrated for 2 min with a descending concentration of graded alcohol (100 − 70%), followed by a 2-min immersion in distilled water. The hydrated tissue sections were stained with 1% hematoxylin for 3 min and then rinsed slowly for 5 min in tap water. The stained slides were dipped in HCl: Ethanol (1:1) solution and kept under running tap water for 5 min to remove the additional stain and further counter-stained with 1% eosin for 30 s. The slides were then dehydrated for 2 min with increasing concentrations of graded alcohol (70–100%). The stained slides were then immersed in xylene for 2 min before being mounted using DPX (Di-butylphthalate Polystyrene Xylene) solution. Finally, images of the stained tissue slices were acquired using a (Leica DM 2000, Germany) microscope.

### Statistical test

GraphPad Prism statistical software (version 8) was used for graphical presentation and data analysis. The regression (R^2^) value for the invasion assay was determined using a nonlinear regression curve. To confirm normal distribution, the Shapiro-Wilk test was conducted. Significance among different experimental groups was assessed using Student’s t-test (two-tailed, unpaired) or the non-parametric Mann-Whitney U test. Statistical significance was indicated by *P ≤ 0.05 and **P ≤ 0.01.

## Results

### Effect of T6SS mediated predation on differential stress tolerance of * C. jejuni*

**Fig. 1:**
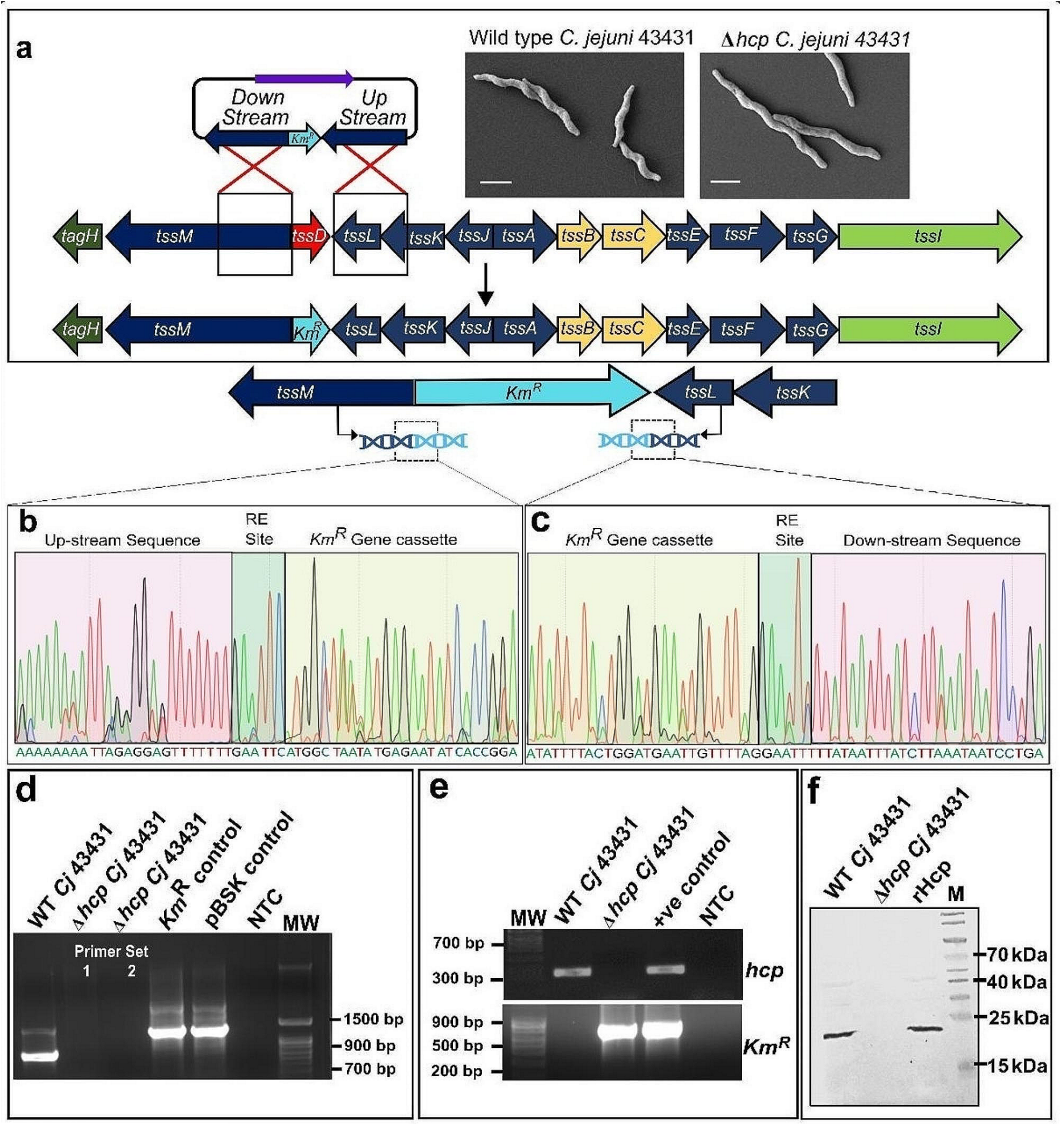
Generation of *hcp* knockout *C. jejuni* mutant (Δ *hcp C. jejuni*). (**a**) A targeted deletion of *hcp* (locus tag NMLJCPGM_01019, size: 516 base pairs) was generated in the *C. jejuni* (ATCC 43431-TGH 9011) by exchanging the gene with a Kanamycin Resistant gene (*Km*^*R*^). Schematic showing homologous recombination between *Km*^*R*^ cassette and the flanking regions of *hcp* in T6SS island of *C. jejuni* genome to generate Δ*hcp C. jejuni* mutant. The FESEM micrographs showed no morphological changes of *C. jejuni* after replacing *hcp* gene with *Km*^*R*^; scale bar: 1 μm. (**b**, **c**) Sequencing chromatogram of the *hcp* knock-out mutant of *C. jejuni* using upstream and downstream primer set of flanking region of *hcp* gene showing the insertion of *Km*^*R*^ in place of *hcp* in genomic DNA of T6SS-positive *C. jejuni* (ATCC 43431-TGH9011). (**d**) PCR analysis of *hcp* deletion and replacement of *hcp* gene with *Km*^*R*^ gene showing no amplification of target gene in the genomic DNA extracted from Δ*hcp C. jejuni* (Lane 2, Lane 3) and amplification of *Km*^*R*^ gene from genomic DNA of Δ*hcp C. jejuni* (Lane 4). Lane 1 and 5 are kept as positive controls, while Lane 6 is no template control (NTC). Here, Lane 1 shows the amplification of the *hcp* gene from the genomic DNA of wild-type *C. jejuni* (TGH 43431), and Lane 5 represents the amplification of the *Km*^*R*^ gene from the *p*BSKII plasmid. (**e**) Transcriptome analysis from WT-*C. jejuni* and Δ*hcp*-*C. jejuni*. Agarose gel image showing the presence of *Km*^*R*^ transcript and the absence of the *hcp* transcript in the *hcp* knockout mutant while in WT-*C. jejuni* detects *hcp* transcript. (**f**) Western blot analysis of the whole cell lysate of *C. jejuni* shows the presence of Hcp protein in *C. jejuni* cell lysate. Detection using an anti-Hcp antibody revealed a protein band at ~ 20 kDa, consistent with Hcp presence. However, the absence of a protein band in the Δ*hcp C. jejuni* strain indicates successful knockout of *hcp.*

We assessed the role of T6SS in bile salt-mediated stress tolerance using two *C. jejuni* reference strains, one with complete T6SS and the other without T6SS. The whole genome sequence analysis revealed 98.05% similarity between T6SS-positive and -negative strains, while the T6SS-positive strain carried 21 T6SS-related genes, which were absent in the T6SS-negative strain (Supplementary Fig. S1a-b). Given that hemolysin-coregulated protein (*hcp*) is a major effector among the 13 genes that encode structural and functional units of the hexameric T6SS channel [37, 49], we generated an *hcp* deletion isogenic mutant of *C. jejuni* strain (Δ*hcp*) to explore the involvement of the T6SS channel in the intracellular transport of bile salt (Fig. 1a). Deletion of *hcp* was confirmed by DNA sequencing (Fig. 1b, c), PCR analysis of genomic DNA (Fig. 1d), amplification from mRNA transcript (Fig. 1e), and Western blot analysis (Fig. 1f).

**Fig. 2:**
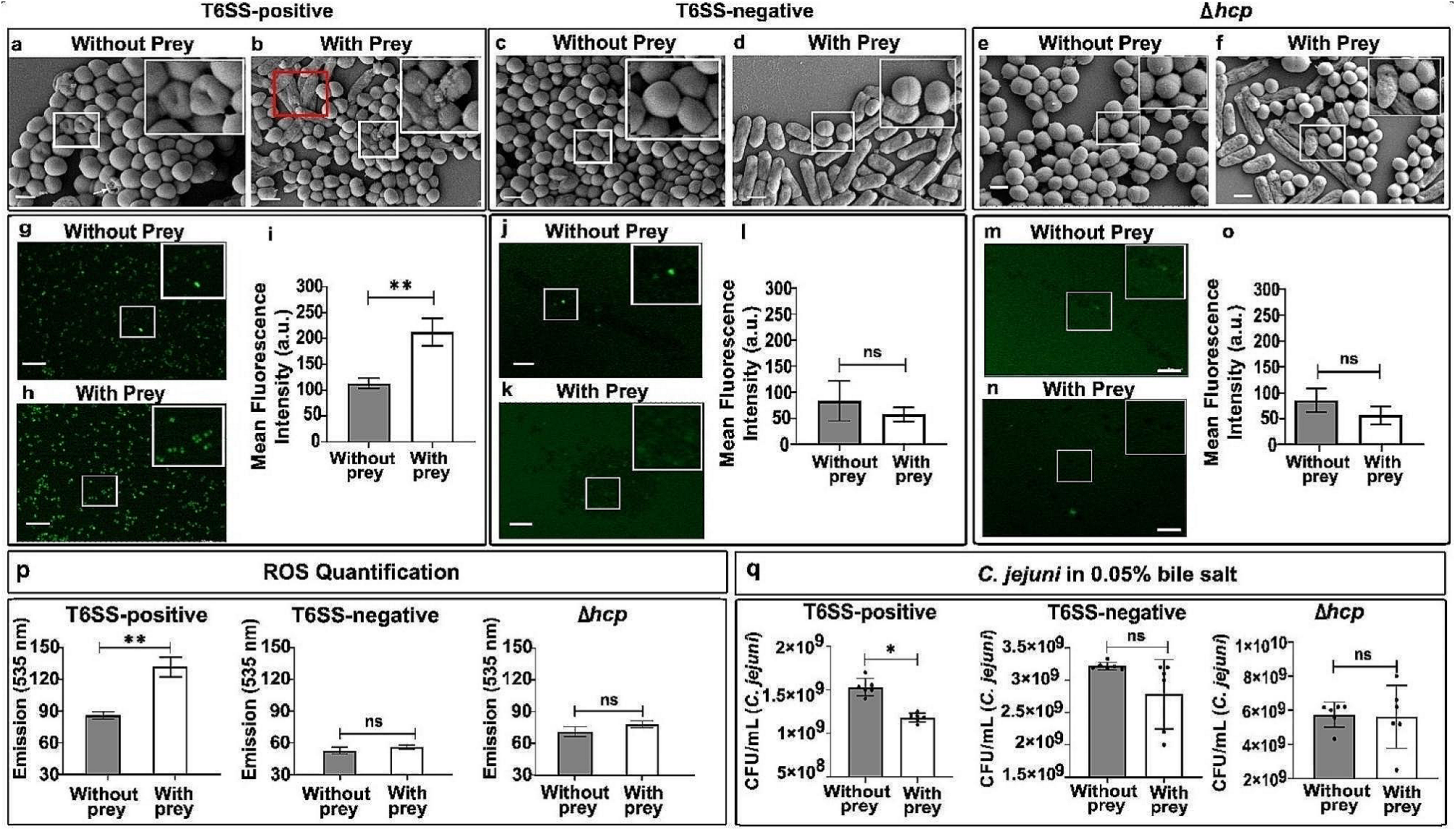
Role of prey (*E. coli*) in differential bile salt tolerance of T6SS-positive, T6SS-negative and Δ*hcp* *C. jejuni.* (**a-f**) FESEM micrographs illustrate clear morphological alterations in *C. jejuni* when exposed to either the presence or absence of prey (*E. coli*). Deflated sac-like morphology of *C. jejuni* was observed, but no such difference was noticed in the case of T6SS-negative *C. jejuni* and Δ*hcp C. jejuni* (see insets). Moreover, prey cell damages were evident (red box) in the presence of *C. jejuni* but not in the case of T6SS-negative *C. jejuni* and Δ*hcp C. jejuni*. Scale bar: 1 μm. (**g-o**) Epifluorescence images showing intracellular ROS generation in *C. jejuni*. H_2_DCFDA-treated *C. jejuni* cells displaying higher fluorescence signals (green) in the case of T6SS-positive *C. jejuni* when the prey is present, indicating a high-level intracellular accumulation of ROS. Little or no fluorescence signal was detected in T6SS-negative cells as well as in the *hcp-*knock out *C. jejuni* (with or without the presence of prey). The fluorescence signal of T6SS-positive *C. jejuni* cells incubated with prey and bile salt exhibited a significantly higher mean fluorescence intensity (MFI) compared to conditions without prey (**i**). Regardless with or without the presence of prey no such elevated MFI was observed in T6SS-negative *C. jejuni *(**l**) and in Δ*hcp C. jejuni* (**o**). Scale bar:10 μm. (**p**) Quantification of total ROS present in *C. jejuni* cells indicates enhanced MFI in T6SS-positive *C. jejuni* when prey bacteria is present. No such difference was noticed for T6SS-negative and Δ*hcp C. jejuni* (*n* = 6). (**q**) When prey was present, the count of T6SS-positive *C. jejuni* colonies (CFU/mL) was notably lower compared to when *C. jejuni* was grown alone. The *C. jejuni* cells were cultured in the growth medium containing bile salt solutions (0.05% w/v). However, there was no alteration in CFU counts observed for T6SS-negative and Δ*hcp C. jejuni* regardless with or without the presence of prey. Error bars depict standard deviation (mean ± SD) (*n* = 6)

Bile salt has been reported to exhibit antibacterial effects, such as disruption of membrane integrity by altering membrane fluidity and permeability, thus affecting the functions of critical membrane proteins [50]. In particular, the FESEM micrographs suggested bile salt-induced damage in the morphology of T6SS-positive *C. jejuni* but not in T6SS-negative cells or Δ*hcp C. jejuni* (Fig. 2a-f; Supplementary Fig. S2, S3, S4; Supplementary Table S4). The critical comparison of FESEM images and the percentage of cell damage among the different experimental groups further suggest that the number of damaged cells (sac-like morphology and even complete lysis/disintegration of T6SS-positive *C. jejuni* cells) was significantly higher (~16.14%) when T6SS-positive *C. jejuni* grown when *E. coli* and bile salt were present compared to when *C. jejuni* grew in bile salt-containing medium but without prey (~0.85%) (Supplementary Fig. S2; Supplementary Table S4).

Such changes in bacterial morphology and physiology are often observed when bacterial cells encounter stress conditions. Since the -OH groups in bile salts are known to cause damage to intercellular organelles and DNA by producing ROS [51], we investigated whether T6SS-positive cells produce a differential amount of intracellular ROS.

The representative apotome images and the mean ROI (region of interest) value for each group indicated that T6SS-positive *C. jejuni*, when *E. coli* and bile salt were present, exhibited significantly higher fluorescence signals (Fig. 2g-i; Supplementary Fig. S5 a, b) than using the Δ*hcp* mutant (Fig. 2m-o; Supplementary Fig. S5 e- f) or T6SS-negative *C. jejuni* (Fig. 2j-l; Supplementary Fig. S5 c, d). Interestingly, we observed higher ROS generation in T6SS-positive cells under bile salt conditions, leading to a loss of bacterial survival when grown together with prey, compared to the case without prey (Fig. 2p). However, when tested across various bile salt concentrations (ranging from 0.05 to 0.125%), the T6SS-negative strain exhibited a higher capacity to withstand stress compared to the T6SS-positive cells (Fig. 2q; Supplementary Fig. S5g). Similar functional attributes in terms of enhanced tolerance to bile salt were detected in the Δ*hcp* mutant, regardless of the presence or absence of prey (Fig. 2q; Supplementary Fig. S5g). Importantly, whether prey was present or absent did not impact the stress tolerance of either the Δ*hcp* mutant or T6SS-negative cells (Fig. 2q). Together, based on our data, it appears that the bile salt susceptibility of *C. jejuni* is linked to Hcp-driven T6SS functionality, which can modulate the prey population.

As an invasive gut bacterium, *C. jejuni* can utilize multiple physiological adjustment systems, including activation of CmeABC efflux pumps, to facilitate self-survival by enduring a harsh gut environment [26]. Notably, the T6SS-positive and-negative strains used in this study carried homologous genes encoding CmeABC efflux pumps. Previously, we reported that *C. jejuni* cells showed less tolerance to predation above the critical bile salt concentration [35]. The present observation further supports that bile salt susceptibility and subsequent ROS generation in *C. jejuni* are direct outcomes of T6SS-mediated predation, indicating that higher bile salt stress leads to the “depletion” of *C. jejuni*. However, the question remains whether effects on *C. jejuni* stress tolerance rely on the intracellular transport of bile salts.

### Enhanced intracellular bile salt influx by T6SS-positive *C.* *jejuni *in the presence of prey

We postulated that the activation of T6SS by prey could result in the intracellular influx of bile salts through the bidirectional contraction of the T6SS apparatus during the secretion of effectors, as previously documented [35]. We anticipated that visualization of bile salt influx could provide direct evidence that a functional T6SS channel can control the dynamics of intracellular transport of bile salts.

**Fig. 3:**
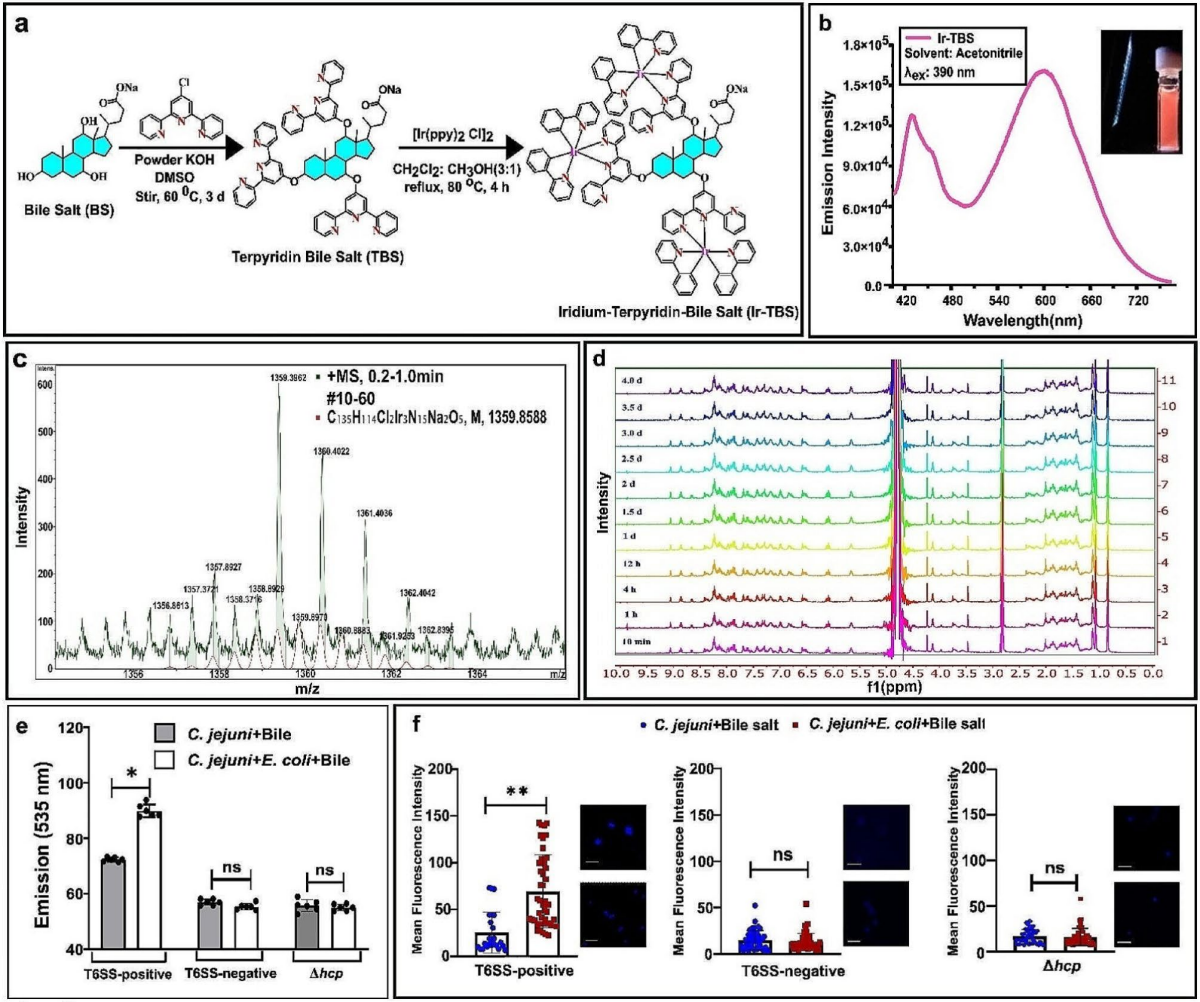
T6SS mediates the intracellular influx of fluorescently labeled bile salt. (**a**) Reaction scheme for synthesizing the iridium (Ir)-conjugated bile salt complex (Ir-TBS). Functionalized bile salt (BS) was reacted with 2,2’:6’,2’’-terpyridine (Tpy), followed by iridium [Ir(ppy)_2_ [Ir = iridium, ppy = 2-phenyl pyridine)] conjugation to generate Ir-TBS complex. Yield: 31 mg (25.5%). (**b**) Photophysical profile of Ir-TBS complex at room temperature in acetonitrile. Spectral data showed that Ir-TBS absorbs at 200–520 nm and emits at λ_max_ (λ_ex_ = 390 nm) = 430 and 600 nm with a shoulder at 436 nm. (**c**) Observed ESI-MS spectra ([M - Cl^−^ + Na^+^]/2 = 1359.3811) of Ir-TBS complex (green) with simulated spectra ([M - Cl^−^ + Na^+^]/2 = 1359.3589) (red) showing the M^2+^ spectral pattern. (**d**) Time-dependent ^1^H NMR showing kinetic stability of Ir-TBS in PBS (pH 7.4) having 0.5% DMSO for 4 days. (**e**) Comparative analysis of emission fluorescence intensity (EFI) of Ir-TBS complexes present in *C. jejuni*. Following incubation of *C. jejuni* cells with Ir-TBS complex for 7 h, the cells were washed and the pellets were processed for spectrophotometry and image analysis. The EFI values indicate higher internalization of Ir-TBS in *C. jejuni* when prey (*E. coli*) was present. No differences were recorded for T6SS-negative and Δ*hcp C. jejuni* (*n* = 6). (**f**) Representative CLSM images of *C. jejuni* cells grown in the presence of prey showed a higher influx of Ir-TBS complex in T6SS-positive *C. jejuni* than in T6SS-negative *C. jejuni* (Scale bar: 2 μm). Further analysis reveals a significant difference in mean fluorescence intensity (MFI) between T6SS-positive *C. jejuni* and both Δ*hcp C. jejuni* and T6SS-negative *C. jejuni* cells under conditions where prey is present. Standard deviations are represented as error bars (mean ± SD) (*n* = 40)

For this, we synthesized iridium conjugated bile salt (Ir-TBS) complex by reacting functionalized bile salt (BS) with 2,2’:6’,2’’-terpyridine (Tpy) molecule (Terpyridine appended bile salt (TBS) followed by conjugation with Ir(ppy)_2_ [Ir = iridium, ppy = 2-phenyl pyridine) (Fig. 3a). The photophysical property of Ir-TBS was confirmed by UV-Vis spectral analysis, while purity, stability, and higher phosphorescence lifetime (µs) of Ir-TBS complex were confirmed by ESI-MS, and stability was confirmed by time-dependent ^1^H NMR (Fig. 3b-d; Supplementary Fig. S6 a-m; Supplementary Table S1). Using this Ir-TBS complex when observed for the fluorescence intensity of Ir, we recorded a significantly higher signal for the T6SS-positive *C. jejuni* compared to the isogenic mutant (Δ*hcp*) and T6SS-negative strain, suggesting that the high-level bile salt influx is mediated by the functional T6SS (Fig. 3e). More interestingly, the Ir signal was enhanced in the case of T6SS-positive *C. jejuni* when prey was present, compared to without prey (Fig. 3f). This could suggest that the prey population triggers functional enhancement of T6SS, leading to a higher bile salt influx.

Since the intracellular accumulation of bile salts is associated with ROS production and subsequent DNA damage [51], the data presented herein suggest the role of T6SS in bile salt sensitivity. Moreover, we showed that the T6SS-mediated predation decreased the self-survival ability of *C. jejuni* under bile salt stress, supporting the notion that T6SS functionality could entail “a cost” for T6SS-positive *C. jejuni* cells when bile salts are present. This atypical feature of the T6SS led us to further test the hypothesis in an in vivo set-up. Given that *C. jejuni* can persistently colonize the chickens gut, leading to a potential source for foodborne transmission to humans, we examined whether these attributes of T6SS could control *C. jejuni* colonization in poultry. Consequently, we comprehensively explored the effect of T6SS-functionality in reducing *C. jejuni* in chickens, using an in vitro setup first and then in an in vivo chickens model.

### T6SS activity can reduce *C.* *jejuni* load in primary chicken embryonic intestinal cells (CEICs)

**Fig. 4:**
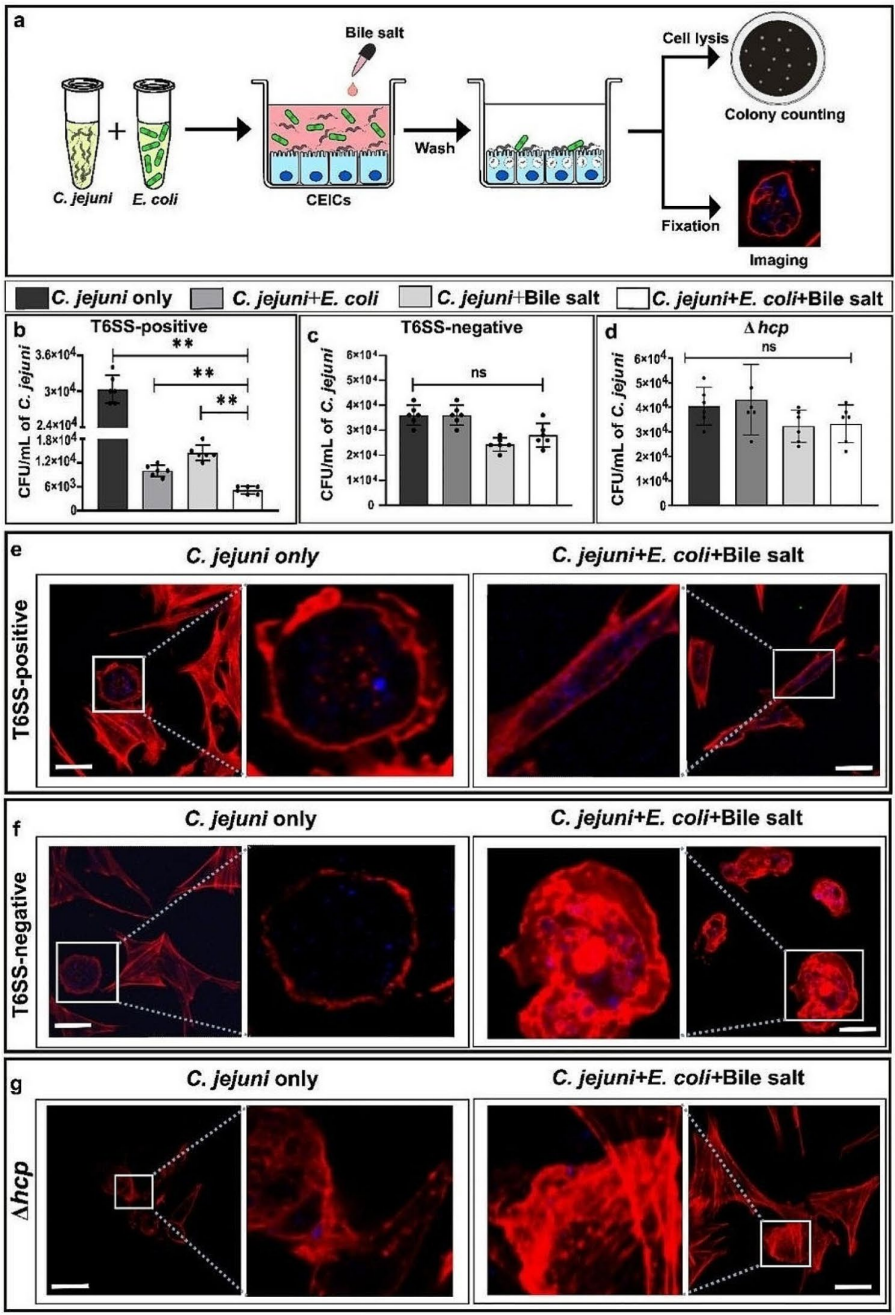
In vitro T6SS activity on *C. jejuni* load in primary chickens embryonic intestinal cells (CEICs). (**a**) Schematic of the in vitro gentamycin protection assay to investigate the effect of T6SS activity on *C. jejuni* adherence and invasion in CEICs. Confluent monolayers of CEICs were co-incubated for 7 h with T6SS-positive, -negative, or Δ*hcp C. jejuni* in the presence of *E. coli* and 0.05% bile salt solution. Subsequently, the cells were washed to eliminate extracellular bacteria and any remaining bile salts in the medium, followed by treatment with gentamycin to remove the adhered bacteria. The invading *C. jejuni* number was calculated (CFU/mL) after lysing the cells using TritonX100. The cells were imaged to visualize cellular changes. (**b, c, d**) The effect of prey on *C. jejuni* load on CEICs. Comparative data indicate a significant decrease in the number of T6SS-positive *C. jejuni* invasions in the presence of prey (*E. coli*) (**b**). Irrespective of the prey, no significant difference in *C. jejuni* population was observed when T6SS-negative (**c**) and Δ*hcp C. jejuni* (**d**) strains were used for infection. (**e, f, g**) Images representing *C. jejuni* (stained with DAPI, shown in blue) invasion of CEICs (cell membrane stained with phalloidin, shown in red) (scale bar: 50 μm) suggest that when prey and bile salt stress (0.05%, w/v) are present, only a small number of T6SS-positive *C. jejuni* are detectable (shown in blue), contrasting with cells infected solely with *C. jejuni* (**e**). Comparison of the images reveals distinct alterations, notably rounded-off cells when prey and bile salt are absent (**e**). Regardless of the involvement of prey and bile salt, CEICs exhibited a rounded shape when infected with T6SS-negative *C. jejuni* (**f**) and Δ*hcp C. jejuni* (**g**). All error bars represent standard deviations, denoted as mean ± SD (*n* = 6)

To determine T6SS functionality under bile salt stress in vitro, with or without the presence of the prey primary CEICs were infected with *C. jejuni* (Fig. 4a). Notably, the comparative abundance (adhered and invaded) of T6SS-positive (WT), T6SS-negative, and Δ*hcp C. jejuni* suggested the lower number of bacterial populations when chicken cells were infected with the T6SS-positive strain with *E. coli* and bile salt (Fig. 4b; Supplementary Fig. S7a). Moreover, irrespective of the prey, *C. jejuni* load in CEICs remained higher for Δ*hcp* and the T6SS-negative strain (Fig. 4c, d; Supplementary Fig. S7 b, c). This was further confirmed by CLSM imaging, which showed that the cellular integrity of CEICs was not affected when prey (*E. coli*) and bile salt were present (Fig. 4e; Supplementary Fig. S8a). Alternatively, the cells exhibited a rounded morphology, accompanied by a notable rise in *C. jejuni* invasion of host cells. Nonetheless, with or without the presence of prey did not markedly influence the interaction of Δ*hcp C. jejuni* or T6SS-negative *C. jejuni* with host cells (Fig. 4f, g; Supplementary Fig. S88b-c).

Taken together, we can infer that the prey-driven activity of T6SS under stress can significantly reduce *C. jejuni* association with host cells. Since our in vitro setting did not consider the complexity of the gut environment, we further tested our hypothesis of T6SS-dependent selective killing of *C. jejuni* in chickens.

### In vivo perturbation of *C. jejuni* self-survival utilizing T6SS mediated predation in chickens

**Fig. 5:**
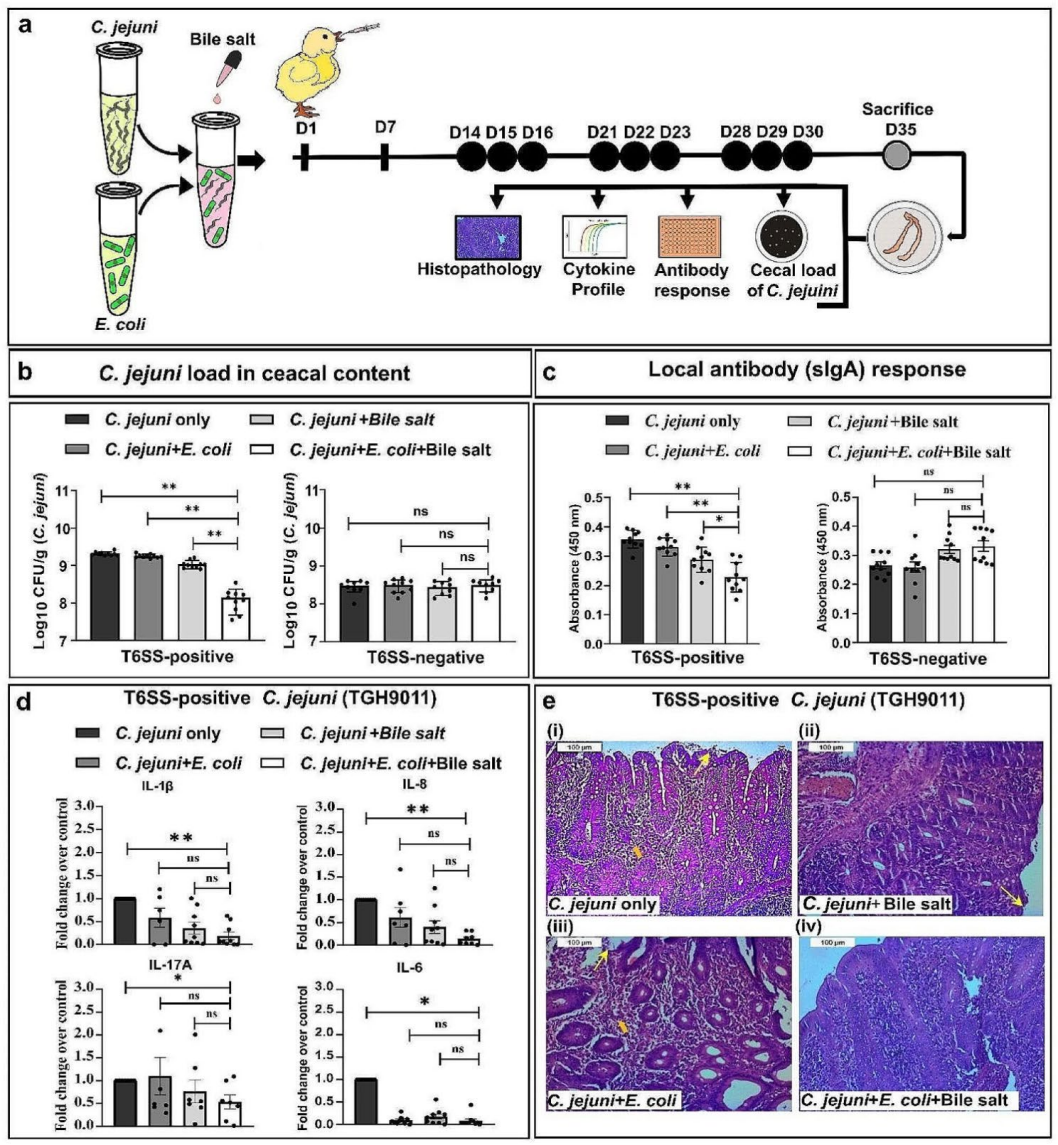
Prey-dependent depletion of *C. jejuni* in chickens maintained on bile salt supplementation. (**a**) Schematic of in vivo chickens feeding trial. After 7 days of acclimatization, birds were maintained in a bile salt (0.2%, w/v) containing diet till day 30. Chickens fed with a normal diet without bile salt were kept in control. From day 14 onwards, experimental birds were fed with either *C. jejuni* or *C. jejuni* and *E. coli* at the indicated time points (black circle). On day 35 (open circle), birds were sacrificed, and cecum, its content, and intestinal lavages were collected for the analysis of bacterial load, anti-*C. jejuni* antibody response, transcriptional profiles of pro-inflammatory cytokines, and cecal tissue histopathological study. The experimental group details were as follows: Group A: *C. jejuni* only (no bile salt); Group B: *C. jejuni* + *E. coli* (no bile salt); Group C: *C. jejuni* + bile salt and Group D: *C. jejuni* + *E. coli* + bile salt. Parallel with this, in vivo feeding with T6SS-negative *C. jejuni* under similar experimental conditions was performed (*n* = 9 birds for each group). (**b**) Cecal load of *C. jejuni* showing effective clearance of *C. jejuni* in Group D birds compared to the other experimental groups. No such difference was observed when T6SS-negative *C. jejuni* was used. (**c**) Comparative analysis of local antibody (sIgA) responses in the intestinal lavage against *C. jejuni* indicates a significantly low level of sIgA titer in the birds belonging to Group D compared to the other experimental groups. Reduced sIgA titer in these birds suggests prey-dependent clearance of T6SS-positive *C. jejuni* in the presence of bile salt. However, no such difference was noticed when the experiment was performed with T6SS-negative *C. jejuni*. (**d**) Pro-inflammatory cytokine gene expression profile of caecal tissue collected from different groups of birds indicates low-level expression of IL-1β, IL-8, IL-17A and IL-6 genes in Group D compared to the other experimental groups. (**e**) Histopathological changes in cecal tissue from different experiment groups. Panels i-iii show higher lesion scores characterized by necrotic lesions (arrow), lymphocytic infiltration (block arrow), disruption in the top layer of epithelium, and unorganized cell boundaries. Panel-iv displays perfectly oriented, continuous, and well-demarked surface epithelium

To explore how the functionality of T6SS of *C. jejuni* impacts its ability to prosper within the chicken gut, a total of 72 day-old chicks were maintained on bile salt supplements. From day 14 onwards, birds were orally administered *C. jejuni* with or without *E. coli* (as prey), at weekly intervals, as shown in Fig. 5a. To define the effect of different feeding regimens on *C. jejuni* clearance, we determined the *C. jejuni* load in freshly collected fecal pellets and cecal content to see the effect of different feeding regimens on *C. jejuni* clearance. Consistent with in vitro results, a significantly low number of *C. jejuni* (~ 1 log) was detected in the group of birds that received T6SS-positive *C. jejuni* along with *E. coli* and bile salts, compared to the groups that received *C. jejuni* only. In contrast, oral delivery of T6SS-negative *C. jejuni*, either alone or with *E. coli*, or with *E. coli* and bile salt, failed to show a noticeable reduction in *C. jejuni* load (Fig. 5b; Supplementary Fig. S9a). The observed differences showed the possibility that if employed under an optimized dose of bile salt, the function of bacterial T6SS can be utilized for purging T6SS-positive *C. jejuni* from the primary host.

Since the intestinal load of *C. jejuni* may correlate with *C. jejuni*-specific antibody response locally, we assessed the secretory IgA (sIgA) antibody titers in the intestinal lavages probed with cell-free lysates of *C. jejuni* by indirect ELISA. Comparing the mean antibody titer of sIgA among different experimental groups, birds administered *C. jejuni* along with *E. coli* and bile salt showed the lowest level of local antibody titer, confirming the previous observation of effective *C. jejuni* clearance in this group. Notably, no substantial difference in the antibody response against *C. jejuni* was detected in birds that received T6SS-negative *C. jejuni* (Fig. 5c; Supplementary Fig. S9b). The persistence of this low-level anti-*C. jejuni* antibodies in birds administered T6SS-positive *C. jejuni* along with prey (*E. coli*), reaffirms the role of functional T6SS in bacterial “depletion.” These outcomes further advocate that the cecal clearance of *C. jejuni* does not require adaptive immunity if T6SS functionality can be exploited in response to bile salt stress.

Although being a commensal, *C. jejuni* resides in the chickens gut, recent findings showed that long-term colonization of *C. jejuni* negatively influences the gut barrier in chickens; particularly, it can lead to a “leaky gut” by an enhanced intestinal permeability [52]. Moreover, persistent colonization of *C. jejuni* aids the translocation of *C. jejuni* to the underlying tissues and the spread of other microbiota, including *E. coli* [53–58]. Therefore, we investigated the pro-inflammatory cytokine gene expression profile and histopathological changes in the cecal tissues to further determine whether the T6SS-dependent cecal clearance of *C. jejuni* positively affects overall gut health.

Given that unregulated inflammation often causes tissue damage, leading to the loss of vital function of intestinal epithelial cells, we assessed the gene expression profiles of IL-6, IL-1β, IL-17A, and IL-8. The birds that received *C. jejuni* along with *E. coli* and bile salt showed a significantly lower grade of pro-inflammatory gene expression than the other experimental groups (Fig. 5d). Furthermore, when checking for histopathological changes in these birds, nearly no inflammatory lesions were detected in the cecal tissue. The cecal mucosa of these birds displayed perfectly oriented, continuous, and well-demarked surface epithelium, lamina propria, and muscular mucosae layer, indicating the maintenance of epithelial cell integrity. Furthermore, the epithelial lining consisted of a simple ciliated columnar type, containing an increased population of goblet cells, suggesting an effective barrier function of the mucosal surfaces (Fig. 5e-iv).

In the case of birds that were not maintained in bile salt supplementation, when challenged with either a combination of *C. jejuni* and *E. coli* or *C. jejuni* only, they showed a significantly higher level of cytokine gene expression accompanied by a moderate to high degree of pathological changes in the cecal tissue. A marked discontinuity was observed in the surface epithelia, focal erosion leading to exposure of the lamina propria to the luminal surface, with a high number of monomorphic and polymorphic lymphocyte accumulations in the lamina propria. Together with a decreased height of glandular epithelia, fewer crypts of Lieberkuhn, and more importantly, visible focal necrosis of the inner circular layer of tunica muscularis were indicative of necrotic inflammation in the cecal tissue (Fig. 5e-i and iii).

In contrast, a low to moderate degree of pro-inflammatory signals in terms of cytokine gene expression was detected in birds that received *C. jejuni* along with bile salt. Distinct inflammatory changes were noted in the lamina propria, with infiltration of monomorphic and polymorphic cells and lymphoid accumulation in the muscularis mucosae layer. Stratification in some sections of the mucosa, with an unusual number of crypts in the lamina propria, was also noticeable. In addition, a bizarre presentation of the muscularis mucosae and degenerative changes in the inner circular layer of the tunica muscularis were visible. Furthermore, the glandular epithelia were found to contain fewer goblet cells, suggesting impaired mucous production and mucosal barrier function in these birds (Fig. 5e-ii).

## Discussion

*Campylobacter* sp. is one of the four major global causes of foodborne diarrheal diseases and is designated a high-priority pathogen on the World Health Organization (WHO) list [59, 60]. *Campylobacter* infection (campylobacteriosis) is estimated to be 4.4–9.3 per 1000 people annually and is a substantial cause of morbidity among children under five years of age [61]. Multiple case studies have documented a notable association between human infection with *C. jejuni* and the ingestion of undercooked poultry contaminated with the bacterium [62, 63]. Hence, poultry products contaminated with *Campylobacter* are regarded as significant contributors to human transmission. Moreover, the prevalence of drug-resistant *Campylobacter* cases has been increasing in many parts of the world, specifically in Low- and Middle-income countries (LMICs). The increasing resistance of *C. jejuni* is presumably due to the injudicious use of antimicrobials in poultry, as supported by several studies [64–66]. Although *C. jejuni* remains commensal in chickens, several recent findings showed that persistent colonization of *C. jejuni* can negatively impact chicken gut health by affecting ion transport, trans-epithelial ion conductance, and increased intestinal permeability [53–58]. Furthermore, given that the metabolic processes of the host are intricately connected with resident gut microflora, the use of antibiotics to target gut pathogens, such as *C. jejuni* can disrupt the gut homeostasis resulting in the overgrowth of potential pathogens [67–69].

Since diverse populations of harmful and beneficial microbes coexist in the chicken gut by sharing resources and space, ideally, alternative antibiotic approaches should be fortified with target-specificity without destabilizing the overall gut homeostasis. Presently, dietary supplementation with probiotics, growth promoters, fecal microbiota transplantation, bacteriophage therapy, and the use of bacteriocins is of note [70–73]. Among them, probiotics, when given in ample quantities, can confer competitive fitness consequences to the host; however, they cannot selectively kill harmful gut pathogens [74]. Therefore, prioritizing the competitive exclusion of potentially harmful gut pathogens while maintaining the balance of gut microbiota, known as eubiosis, is preferable. In the search for alternative strategies for prophylaxis and control of gut pathogens, in the present study, we relied on the unique and atypical functionality of the bacterial intrinsic secretion system, T6SS of *C. jejuni*.

As a dynamic secretion system, T6SS functions by cycles of assembly, contraction, and disassembly to deliver various effectors targeting prey cells [10–14, 75–79]. Moreover, *in silico* analysis further revealed the genetic diversity of *C. jejuni* T6SS and effector-immunity pair which provide a framework for studying T6SS functionality [80, 81]. In line with these suppositions, using the in vitro “two species competition” model we showed that the T6SS activity of *C. jejuni* can effectively kill target bacteria (*E. coli*) and may incur “a cost” during bacterial competition in the presence of environmental stress [35]. However, it remains unclear how the target-driven T6SS functionality prevails in a polymicrobial complex gut environment.

To understand the mechanism of prey-driven T6SS activity under environmental stress, such as bile salt, *hcp* mutant (Δ*hcp*) with an isogenic background of a T6SS-positive *C. jejuni* strain was created. Further to confirm the effect of bile salt during T6SS activity, we showed a high intracellular bile salt influx in T6SS-positive *C. jejuni* compared to the isogenic mutant using fluorophore-tagged bile salt. We propose that higher accumulation of bile salt during predation and subsequent oxidative damage to the DNA caused the morphological changes of *C. jejuni* and the negative effect of T6SS on its self-survival in the presence of *E. coli*. Our real-time tracking of the Ir-conjugated bile salt complex also advocates that *C. jejuni* T6SS can act as a ‘cell-puncturing device’ for effectors on the one hand and a target-driven ‘import system’ on the other hand, possibly by altering the envelope permeability barrier and promoting the intracellular transport of bile salt from the extracellular milieu [14, 35].

While intestinal bacteria utilize bile salts as nutrients, environmental signals, and electron acceptors, *C. jejuni* can flourish in bile salts due to various mechanisms, including the CmeABC efflux pump [26, 82]. In contrast, prey-induced perturbation of bile salt tolerance of T6SS-positive *C. jejuni* (but not in Δ*hcp* or T6SS-negative cells) further strengthens our hypothesis that the dynamic and target-driven T6SS activity may exhibit bidirectional effector functions, leading to higher bile salt transport in the presence of prey.

These attributes of T6SS functionality raise the possibility of using stress-induced “depletion” of *C. jejuni* under in vivo conditions. Since *C. jejuni* is known to be commensal in chickens, we tested whether T6SS-dependent depletion of *C. jejuni* during predation can perturb *C. jejuni* association in chickens. We first confirmed that under in vitro conditions, adhered and invaded *C. jejuni* populations were substantially reduced when primary chicken embryonic intestinal cells (CEICs) were grown with *C. jejuni* and its prey (*E. coli*).

Based on the in vitro results, we next aimed for in vivo dysbiosis of *C. jejuni* in commercial broiler chickens and showed that the intestinal colonization of *C. jejuni* can be reduced by oral administration of *C. jejuni* with its prey and bile salt supplements. Typically, bile acids are produced by the liver and expelled into the digestive tract by the gallbladder when required. Additionally, in their role in digestion, bile salts help in maintaining gut homeostasis by determining the microbial ecology of the intestine [83, 84]. Moreover, bile salts demonstrate antibacterial effects by disrupting bacterial membranes, altering protein structures, binding with iron and calcium ions, inducing oxidative damage to DNA, and regulating the expression of eukaryotic genes associated with host defense and immunity [85]. Previous studies, including ours, have described a generic function of T6SS in competing with neighboring bacteria during interbacterial competition [9, 86]. In contrast, our in vivo trials showed that stress-induced T6SS activities could antagonize *C. jejuni* colonization in a complex gut environment, supported by positive host responses such as reduced anti-*C. jejuni* antibodies in the intestine, accompanied by low-grade pro-inflammatory cytokine gene expression. Since intestinal inflammation can perpetuate mild-to-severe pathological changes in the intestinal mucosa, chronic infection with *C. jejuni* can result in compromised epithelial barrier function in chickens [53, 87, 88]. To this end, histopathological assessment of chicken cecal tissue also suggests that administering *C. jejuni* along with prey and bile salt can maintain intestinal morphology. Furthermore, we ensured that the chickens were free from *C. jejuni* colonization from day 1 to day 7 (before the feeding trial started) by molecular and cultural screening of raw fecal samples.

However, as a dynamic and specialized secretion system, despite the cost for “resource sharing”, bacterial T6SS functionality may not incur a cost for intra-species competition, as reported recently [14], possibly due to the intrinsic defense via immunity proteins. In contrast, our data, supported by others, suggest that T6SS activity during intra- or inter-species interaction could be costly under specific conditions and in the presence of certain bacteria [22, 35, 89, 90].

Since infection with T6SS-positive *C. jejuni* often causes bloody diarrhea more commonly than T6SS-negative *C. jejuni* [91], our key objective was to selectively target the T6SS-positive *C. jejuni* in chickens as a measure to restrict their human transmission. Based on our study, ideally, exogenous administration of an optimal amount of bile salt should drive the selective depletion of T6SS-positive *C. jejuni* in the polymicrobial gut environment, however, studying “two species” competition in an in vivo setup is challenging. Nevertheless, we provide adequate evidence of improved gut health by demonstrating a marked reduction in T6SS-positive *C.jejuni* in chickens compared to T6SS-negative led us to conclude that careful tuning of T6SS functionality of *C. jejuni* can result in more favorable outcomes.

In fact, available literature suggests that even a 2–3 log reduction in the cecal load of *C. jejuni* would reduce approximately 58% of human infections via foodborne transmission, which is considered a therapeutic benchmark from the risk of zoonotic transmission point of view [92]. The influence of gut microbiome on host adaptability primarily hinges on the symbiotic relationship among gut microbes, the nutritional status of the host, and bacterial resilience against natural adversaries [93, 94]. Generally, the evolutionary adaption of the T6SS secretion system provides a unique survival advantage over other bacteria within the same niche [6]. Thus, functional T6SS can have both advantages and disadvantages, depending on the composition of the gut environment.

## Conclusion

Our findings suggest that leveraging the functionality of the T6SS in the intricate gut environment could serve as a potential strategy to mitigate the persistent colonization of T6SS-positive *C. jejuni* in chickens. We suggest that harnessing the prey-driven T6SS of *C. jejuni* could lead to the development of novel alimentary formulations that protect birds from various enteric pathogens carrying T6SS. While the acquisition of bile tolerance is a natural process for major gut microbiota, adapting to bile salts may impact the probiotic characteristics of beneficial gut microbes. Thus, additional research is necessary to determine the optimal form and dosage for the external application of bile salts.

### Electronic supplementary material

Below is the link to the electronic supplementary material.

Supplementary Material 1

## Data Availability

No datasets were generated or analysed during the current study.
