# Supplementary material for: Selective depletion of Campylobacter jejuni via T6SS dependent functionality: an approach for improving chickens gut health
Source: Gut Pathog. 2024 Jul 12;16:38. doi: 10.1186/s13099-024-00628-6 (PMC11245787; doi:10.1186/s13099-024-00628-6)
Supplement: Supplementary file 1 — Supplementary Material 1 [file 13099_2024_628_MOESM1_ESM.pdf]

Supporting Information

Selective depletion of *C. jejuni* via T6SS dependent functionality: an approach for improving chicken gut health

Subhadeep Gupta[1], Prakash Biswas[1], Bishnu Das[2], Samiran Mondal[3,] Parna Gupta[2], Dipjyoti Das[1*] and Amirul Islam Mallick[1*]

[1]Department of Biological Sciences, Indian Institute of Science Education and Research Kolkata, Mohanpur, Nadia, West Bengal-741246, India

[2]Department of Chemical Sciences, Indian Institute of Science Education and Research Kolkata, Mohanpur, Nadia, West Bengal-741246, India

[3]Department of Veterinary Pathology, West Bengal University of Animal and Fishery Sciences, Kolkata, West Bengal, 700037, India

Supplementary Table S1: Photo-physical characterization of TBS and Ir-TBS complex at 25 °C

TBS								
λ_{abs}(nm)	λ_{em}(nm)	ϕ(%)	λ_{max} (nm)	τ_1(ns)	τ_2 (ns)	τ_3(ns)	τ_{av}(ns)	χ^2
309(0.180), 297(0.430), 276(0.831), 241(0.866), 212(0.527)	327, 341, 400	3.54	341	0.36	1.70	3.46	1.89	1.03
			400	2.00	0.35	6.36	3.58	1.03
**Ir-TBS**								
λ_{abs}(nm)	λ_{em}(nm)	ϕ(%)	λ_{max} (nm)	τ_1(μs)	τ_2(μs)	τ_3(μs)	τ_{av} (μs)	χ^2
473(0.014), 437(0.030), 407(0.070), 369(0.137), 306(0.525), 252(1.000), 208(1.357)	430, 456, 600	1.64	430	7.18	0.83		1.01	0.99
			600	0.73	6.34		0.81	0.99

Supplementary Table S2: Composition of poultry feed used in the present study [1].

Ingredients	Pre-starter feed (0 to 10 days)	Starter feed (11 to 35 days)
**Maize**	550	590
**Soya Deoiled cake***	390	340
**Oil**	16.5	27
**Dicalcium Phosphate**	12	12
**Line Stone Powder**	16	16
**Trace Mineral (Manganese, Zinc, Iron, Iodine, Copper, Cobalt)**	2	2
**Salt**	2.5	2.5
**Sodium bicarbonate**	1.5	1.5
**Choline Chloride**	0.5	0.5
**Lysine**	2.5	2
**D.L. Methionine**	2.8	2.7
**Toxin Binder**	1	1
**Emulsifier**	0.25	0.25
**Threonine**	0.15	0.15
**Phytase 5000**	0.1	0.1
**Total**	999.9	999.8
Bile salt solution (mixture of sodium cholate and sodium deoxycholate, 50 %-5 0%, w/w, HiMedia, India) was orally administered at indicated time points (Fig.4A)		

Supplementary Table S3: List of primers used in this study.

Sl NO.	Gene	Sequence (5’-3’)	Reference
1.	*hcp* Up Stream	F-CGGGCGCTCGAGATAATTAAGGGTAAA TTTTTTGTTTTTAAATC	For this Study
2.		R-AGCCGCGAATTCAAAAAAACTCCTTTAA TTTTTTTTAACATTC	
3.	*hcp* Down Stream	F-TTGGACGAATTCTTATAATTTATCTTAAA TAATCCTGACTAA	For This study
4.		R-TGCTATCGGATCCTTGCCACATCTTTAA AACCGGTTTATAAG	
5.	*Km*^{R}	F-CGCGAATTCATGGCTAAAATGAGA ATATCA	For This study
6.		R-CGCGAATTCCTAAAACAATTCATC CAGTAA	
7.	*hcp* detection Primer Set 1	F-ATAGGATCCATGGCTGAACCAGCGTT TATA	For this Study
8.		R-CGCGAATTCTAGCAAAGGCACAGATTT	
9.	*hcp* detection Primer Set 2	F- ATAGGATCCCCAACAGTCGAAG TTCATTGG	For This study
10.		R- ATAAAGCTTGATGATTGGAGAGA GGGCAAA	
11.	Chicken β-actin	F- GAGAAATTGTGCGTGACATCA	[2]
12.		R- CCTGAACCTCTCATTGCCA	
13.	Chicken IL 1β	F-AGTGAGGCTCAACATTGCGCTGTA	For this Study
14.		R-TAGAAGATGAAGCGGGTCAGCTCG	
15.	Chicken IL 17	F-ATTCCAGGTGCGTGAACTCGGC	For This study
16.		R-GTGCAGCCCACGGTGATCATTTTC	
17.	Chicken IL 6	F- GGAACAACCTCAACCTGCCCAAGG	[2]
18.		R- CCAGGTGCTTTGTGCTGTAGCAC	
19.	Chicken IL 8	F- CAGCTGCTCTGTCGCAAGG	[2]
20.		R- GAGCAGTGGGGTCCAAGCACAC	

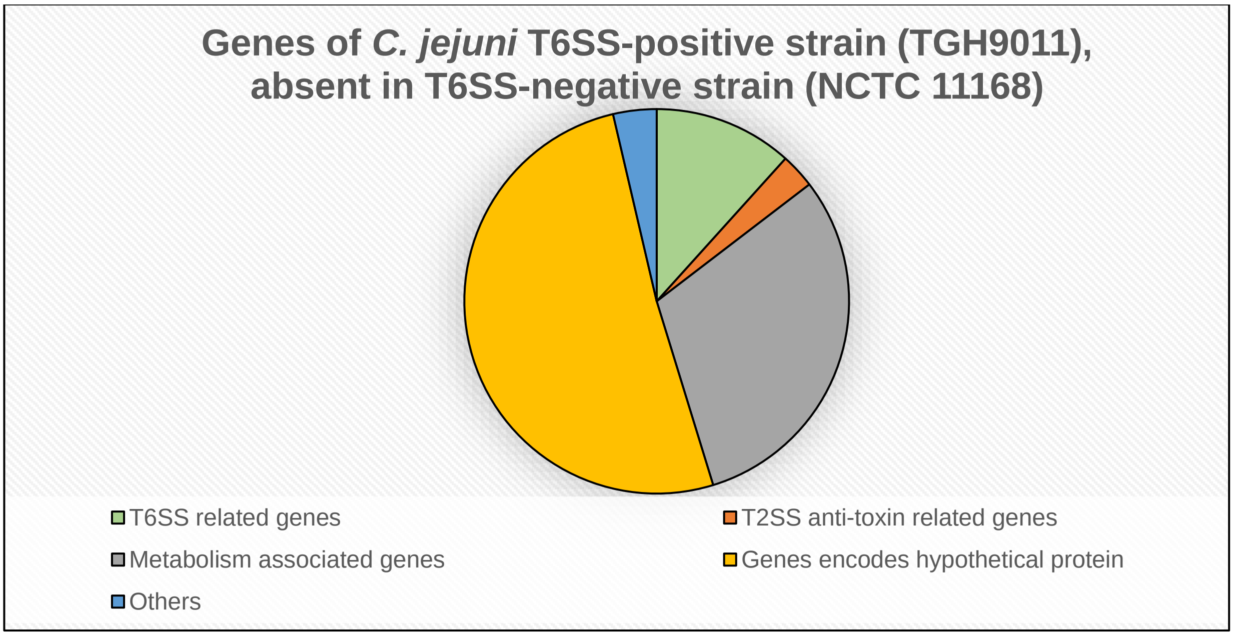

a.

Gene Family	Function	Number of proteins
T6SS related genes	T6SS assembly and functionality	16
T2SS Anti-toxin-related genes	Defense against T2SS	4
Genes encoding metabolism-associated proteins	Protein metabolism	13
	Nucleotide Metabolism	13
	Carbohydrate Metabolism	12
	Lipid Metabolism	4
Genes encoding hypothetical proteins	Unknown Function	70
Others	NAD^+-glycohydrolase activity	1
	Conjugal transfer protein	1
	Na^+-independent uptake of cationic amino acids	1
	Motility associated gene	1
	Autotransporter	1

b.

Fig. S1: Comprehensive sequence analysis of T6SS-positive (TGH9011) and T6SS-negative (NCTC 11168).

- **a.** Pie chart showing T6SS-positive C. jejuni strain genes absent in T6SS-negative *C. jejuni* strain.
- **b.** Table showing gene family and their encoded proteins present in T6SS-positive *C. jejuni* strain but absent in T6SS-negative *C. jejuni* strain.

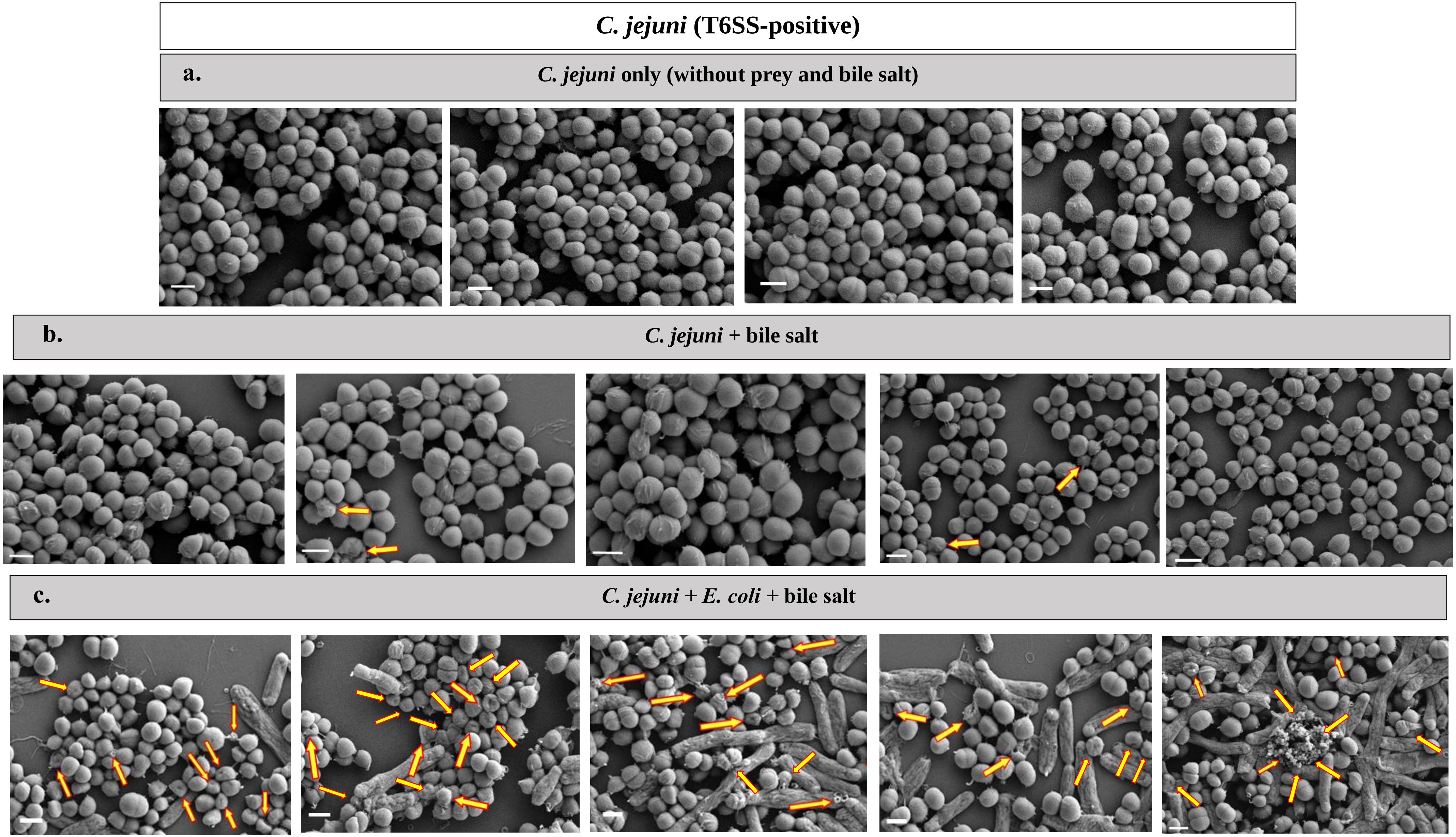

Fig. S2: FESEM micrographs showing in the presence of prey (*E. coli*) and bile salt **(panel c),** T6SS-positive *C. jejuni* exhibits extensive damage (shrank, deflated sac-like morphology and complete disintegration) compared to when T6SS-positive *C. jejuni* grown in the presence of bile salt but without prey **(panel b).** Only a few shrank, deflated sac-like changes are visible in the latter case. Control cells (**panel a**, without prey and bile salt) show no changes. The yellow arrow indicates morphological damage to *C. jejuni* cells. Scale bar: 1 µm.

C. jejuni (T6SS-negative)

a. ***C. jejuni* only (without prey and bile salt)**

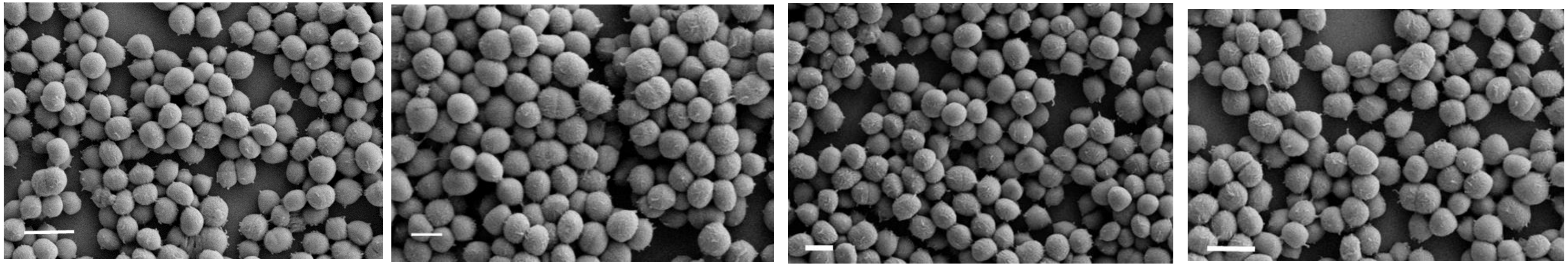

b. ***C. jejuni* + bile salt**

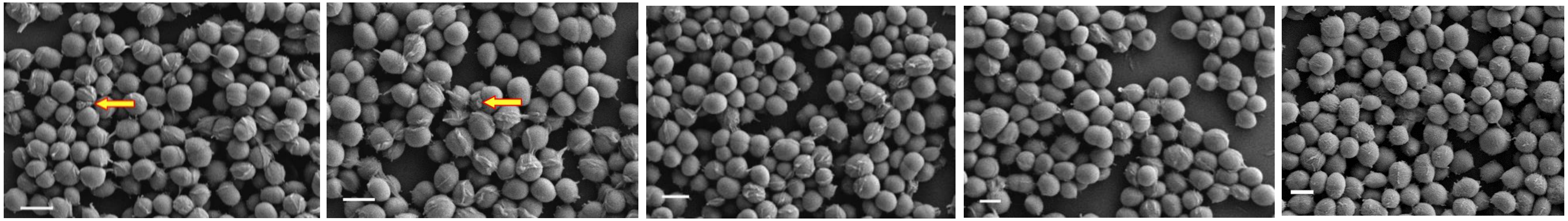

c. ***C. jejuni* + *E. coli* + bile salt**

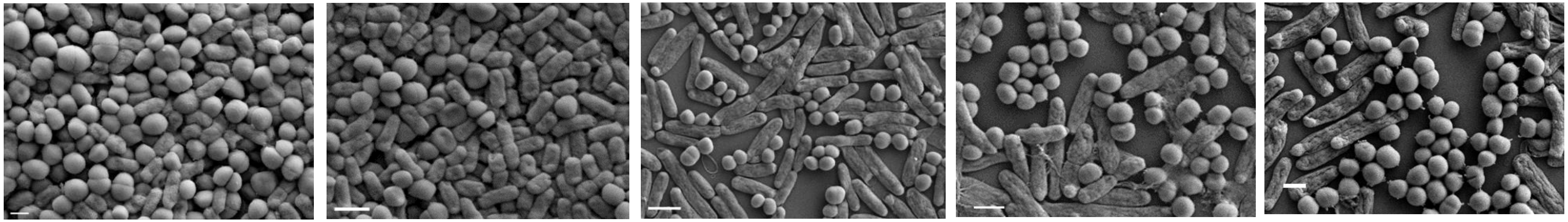

Fig. S3: FESEM micrographs showing in the presence or absence of prey (*E. coli*) or bile salt**,** T6SS-negative *C. jejuni* exhibits no visible changes. Only a few cells showing some minor changes in the morphology of T6SS-negative *C. jejuni* grown in the presence of bile salt, which could be due to the natural antimicrobial effect of bile salt. Yellow arrow indicates morphological damage to *C. jejuni* cells. Scale bar: 1 µm.

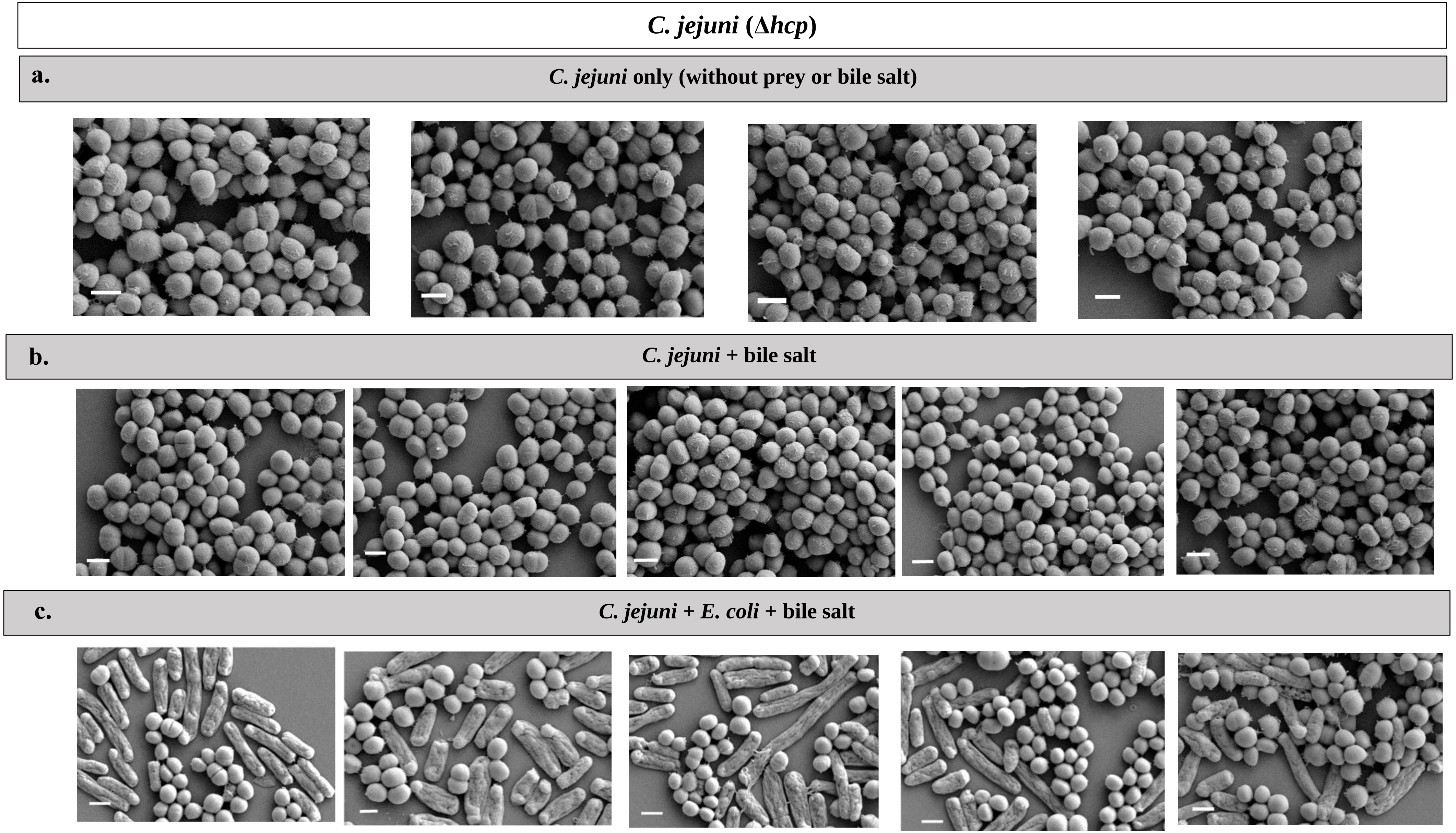

Fig. S4: FESEM micrographs showing in the presence or absence of prey (*E. coli*) or bile salt**, Δ*hcp C. jejuni*** exhibits no visible changes. Scale bar: 1 µm.

Supplementary Table S4: Quantitative analysis of morphological damages caused to *C. jejuni* cells grown under different conditions. A total of 5 FESEM images per group were manually scanned, and the number of damaged cells was counted for each field.

**Groups**	**FESEM**	**T6SS-positive *C. jejuni***						**T6SS-negative *C. jejuni***						**Δ*hcp C. jejuni***					
		**Number of fields**					**Percentage of cell damage**	**Number of fields**					**Percentage of cell damage**	**Number of fields**					**Percentage of cell damage**
		**1**	**2**	**3**	**4**	**5**		**1**	**2**	**3**	**4**	**5**		**1**	**2**	**3**	**4**	**5**	
***C. jejuni* only**	**Total Cell counted cell/ Field**	101	96	91	97	-	**0**	107	97	119	98	-	**0**	93	90	99	77	-	**0**
	**Damaged cells**	0	0	0	0	-		0	0	0	0	-		0	0	0	0	-	
	**Percentage of Damage**	0	0	0	0	-		0	0	0	0	-		0	0	0	0	-	
***C. jejuni*+ bile salt**	**Total Cell counted cell/ Field**	99	70	104	86	110	**0.85**	106	107	100	106	81	**0.47**	101	121	96	96	103	**0**
	**Damaged cells**	0	2	2	0	0		1	0	1	0	0		0	0	0	0	0	
	**Percentage of Damage**	0	2.857	1.923	0	0		0.9	0	1	0	0		0	0	0	0	0	
***C. jejuni*+ *E. coli*+ bile salt**	**Total Cell counted cell/ Field**	37	66	87	73	55	**16.14**	62	41	37	57	65	**0**	31	29	60	34	38	**0**
	**Damaged cells**	9	14	8	7	9		0	0	0	0	0		0	0	0	0	0	
	**Percentage of Damage**	24.4	21.2	9.2	9.6	16.3		0	0	0	0	0		0	0	0	0	0	

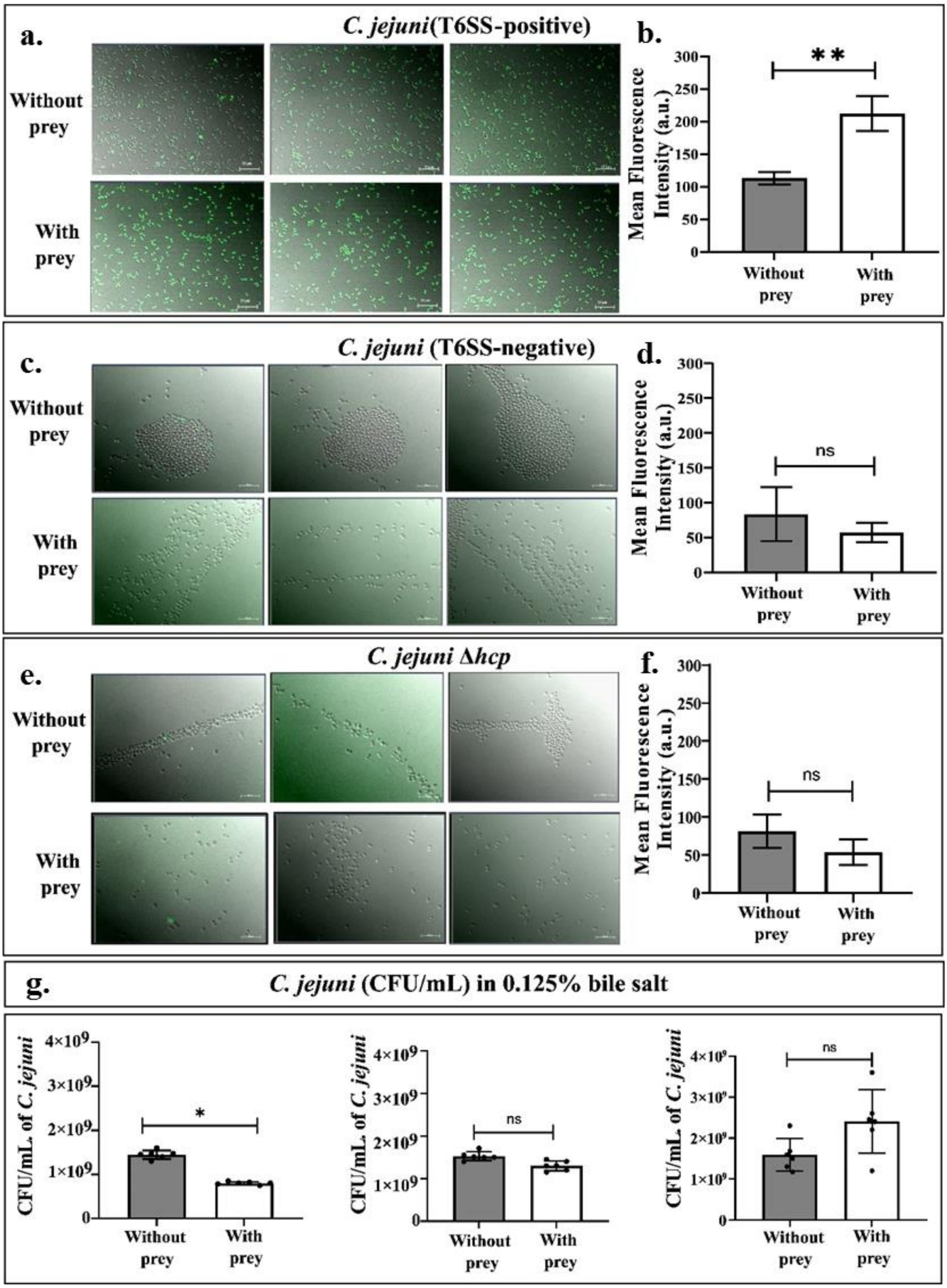

Fig. S5: Role of prey (*E. coli*) in differential bile salt tolerance of T6SS-positive and T6SS-negative and Δ*hcp C. jejuni*. (a-f) The epifluorescence images (**a, c, e**) and respective ROI values (b, d, f) indicate that H_2DCFDA-treated T6SS-positive *C. jejuni* cells in the presence of *E. coli* and bile salt exhibited significantly higher fluorescence signals (green) (a, b) than other control groups. Little or no fluorescence signal was detected in T6SS-negative (c, d) and Δ*hcp* (e, f) cells (in the presence or absence of prey). Scale bar:10 µm. **(g)** In the presence of prey, the number of (CFU/mL) *C. jejuni* colonies was found to be significantly lower than when *C. jejuni* was grown alone. The *C. jejuni* cells were cultured in the growth medium containing bile salt solutions (0.125% w/v). However, no change in CFU counts was observed for T6SS-negative *C. jejuni* and Δ*hcp* in the presence or absence of prey. All error bars represent standard deviation (mean ± SD) (n = 6).

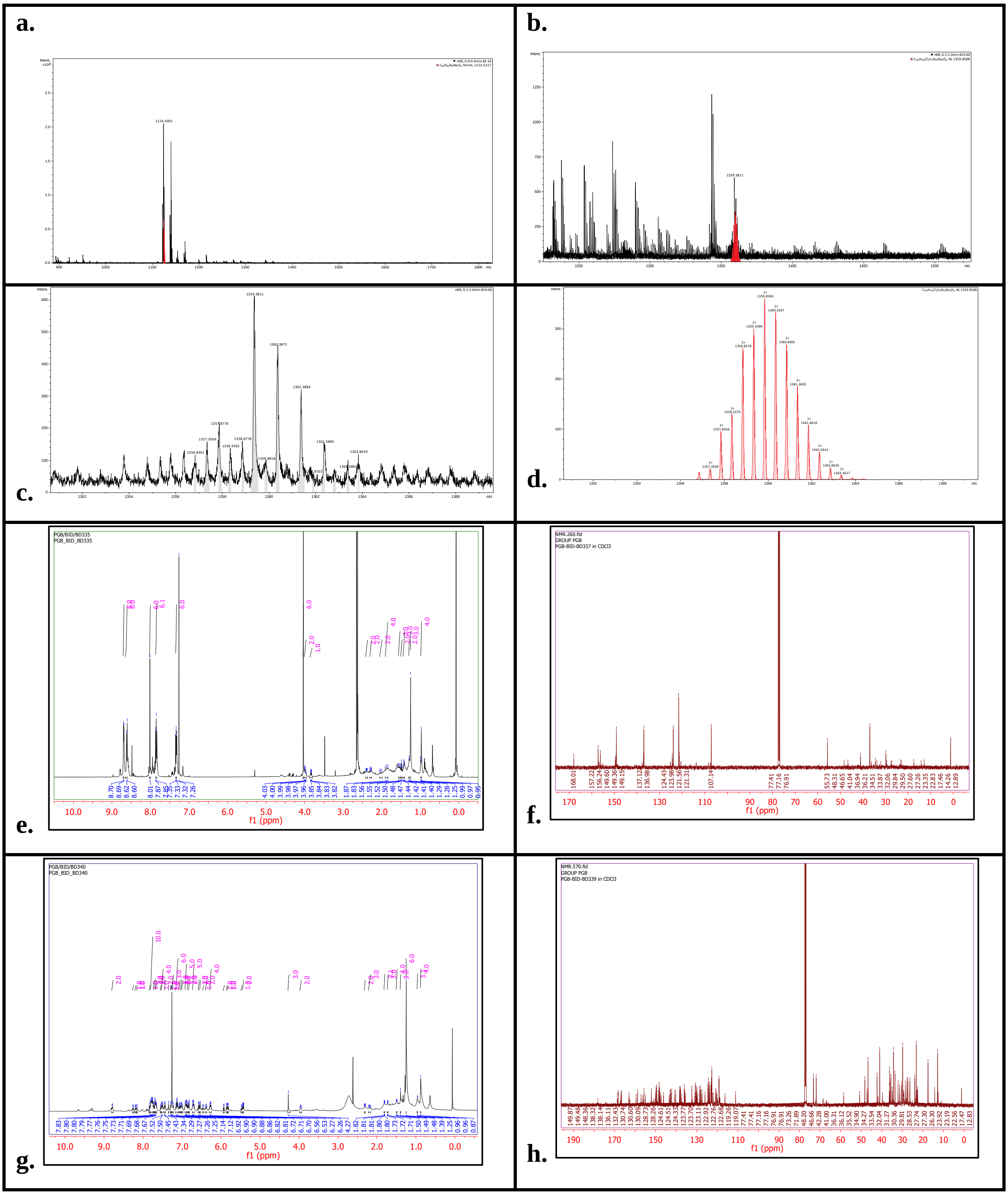
a.
b.
c.
d.
e.
PGB/BID/BD335
PGB_BID_BD335
f1 (ppm)
f.
NMR.260.fid
GROUP PGB
PGB-BID-BD337 in CDCl3
f1 (ppm)
g.
PGB/BID/BD340
PGB_BID_BD340
f1 (ppm)
h.
NMR.570.fid
GROUP PGB
PGB-BID-BD339 in CDCl3
f1 (ppm)

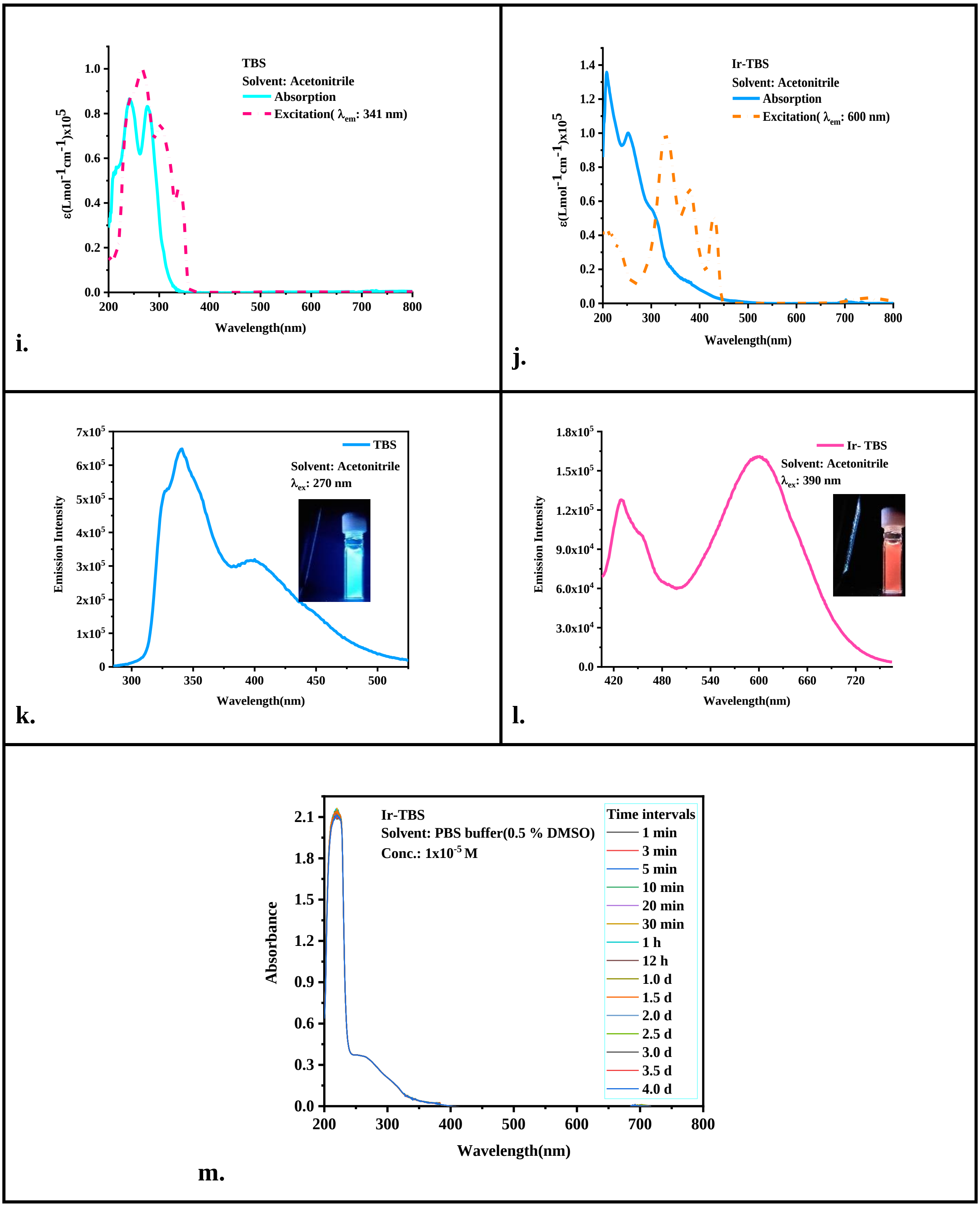

Fig. S6: Analysis of elemental, structural characterization and photo-physical properties of TBS and Ir-TBS complex.

a. Observed ESI-MS of TBS: [M + H^+] =1124.5001 (Exact: 1124.5157)

b. Observed ESI-MS of Ir-TBS: [M - Cl^- + Na^+]/2 = 1359.3811 (Exact: 1359.3589) showing M^{2+} spectral pattern of Ir-TBS complex.

(c-d) Observed (c) and Simulated (d) ESI-MS pattern of Ir-TBS showing the observed isotopic ESI-MS showing an M2+ ionic pattern of Ir-TBS, which is completely matching with the simulated M2+ pattern.

e. ^{1}**H NMR of TBS (500 MHz, $CDCl_3$) δ(ppm):** 8.69(6H, d, J= 4.36); 8.61(6H, d, J= 8.08); 8.01(6H, s); 7.85(6H, t, J= 7.64); 7.33(6H, t, J= 6.36); 4.03(6H, s); 4.00-3.95(2H, m); 3.84-3.81(1H,

m); 2.41-2.36(2H, m); 2.30-2.25(2H, m); 2.02(2H, t, J= 11.48); 1.87(4H, t, J= 13.8); 1.56-1.52(3H, m); 1.48(2H, t, J= 6.92); 1.44-1.40(3H, m); 1.28((2H, d, J= 3.92); 1.24(3H, S); 0.97(4H, t, J= 7.56)

f. ^{13}C NMR of TBS (500 MHz, $CDCl_3$) δ (ppm): 178.25, 168.01, 157.22, 156.88, 156.24, 155.16, 149.60, 149.15, 137.78, 136.98, 125.27, 124.43, 123.98, 121.56, 120.61, 113.82, 108.43, 107.62, 107.14, 55.73, 48.31, 46.65,41.07, 36.97, 36.94, 36.19, 34.51, 33.87, 29.84, 29.50, 27.26, 23.35, 22.83, 17.46, 14.26, 12.89

g. ^{1}H NMR of Ir-TBS (500 MHz, $CDCl_3$) δ (ppm): 8.78(2H, d, J= 5.28); 8.25(1H, d, J= 7.84); 8.19(1H, d, J= 4.52); 8.15(1H, d, J= 4.08); 8.73(1H, d, J= 8.04); 7.80-7.75(10H, m); 7.72(2H, d, J= 7.28); 7.68(2H, d, J= 4.84); 7.66-7.64(2H, m); 7.54(1H, d, J= 7.12); 7.50(4H, t, J= 5.76); 7.43(2H, d, J= 5.76); 7.34(1H, s); 7.28(1H, s); 7.27(1H, s); 7.24-7.22(3H, m); 7.15-7.10(6H, m); 7.07-7.05(2H, m); 7.02-6.98(2H, m); 6.91-6.88(5H, m); 6.85(2H, d, J= 7.48); 6.81(2H, m); 6.73-6.68(5H, m); 6.57(2H, d, J= 7.12); 6.53(2H, d, J= 6.40); 6.46(1H, d, J= 7.56); 6.38(2H, t, J= 7.92); 6.28-6.25(4H, m); 5.92(1H, d, J= 7.68); 5.84(1H, d, J= 7.60); 5.81(1H, d, J= 7.64); 5.47(1H, d, J= 7.60); 5.44-5.41(2H, dd, J= 4.92); 4.27(3H, s); 3.97-3.94(2H, br); 2.31-2.22(2H, br); 2.21(3H, br); 1.81-1.79(3H, br); 1.73-1.71(3H, m); 1.50-1.48(4H, m); 1.39(3H, s); 1.24(6H, br); 0.96-0.95(3H, br)); 0.87(4H, br)

h. ^{13}C NMR of Ir-TBS (500 MHz, $CDCl_3$) δ (ppm): 178.16, 166.69, 163.05, 159.68, 158.86, 155.68, 152.13, 149.48, 148.36, 146.19, 142.26, 140.27, 138.32, 137.36, 136.16, 132.43, 130.58, 129.97, 128.73, 128.26, 127.50, 126.01, 124.33, 123.77, 123.11, 122.68, 120.80, 119.26, 116.27, 111.13, 73.26, 71.89, 48.20, 46.66, 42.28, 41.05, 36.12, 35.52, 34.27, 33.54, 32.04, 29.81, 28.53, 27.30, 26.30, 23.92, 23.19, 22.56, 17.47, 14.24, 12.83

(i-j) UV-Vis and excitation Spectra of TBS and Ir-TBS complex showing the TBS and Ir-TBS absorption in the range 200-370 nm and 200-520 nm, respectively.

(k-l) Emission Spectra of TBS and Ir-TBS showing the emission of at λ_{max} (λ_{ex} = 270 nm) 341, 400 nm with a shoulder at 327 nm for TBS (i) and λ_{max} (λ_{ex} = 390 nm) = 430, 600 nm with a shoulder at 436 nm for Ir-TBS.

m. The time-dependent UV-Vis spectra in PBS with 0.5% DMSO showing kinetic stability of Ir-TBS complex.

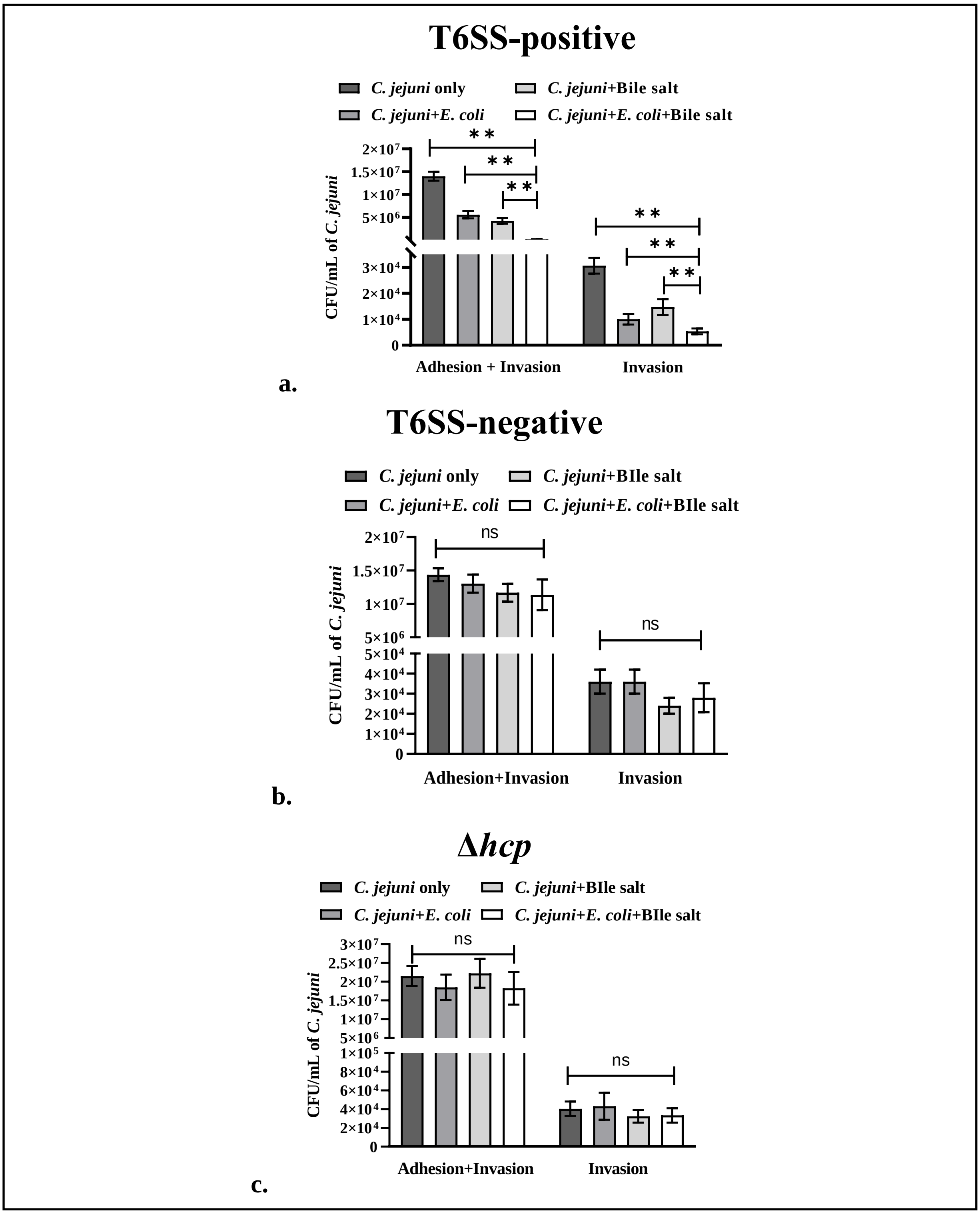

Fig. S7: Effect of prey on cecal load of *C. jejuni* in primary CEICs. Comparative analysis of cecal load of *C. jejuni* indicates a significant reduction in T6SS-positive *C. jejuni* number in the presence of prey (*E. coli*) and bile salt (**a**). However, in the presence or absence of prey, no such difference can be observed in the case of T6SS-negative (b) or Δ*hcp* (c) *C. jejuni*.

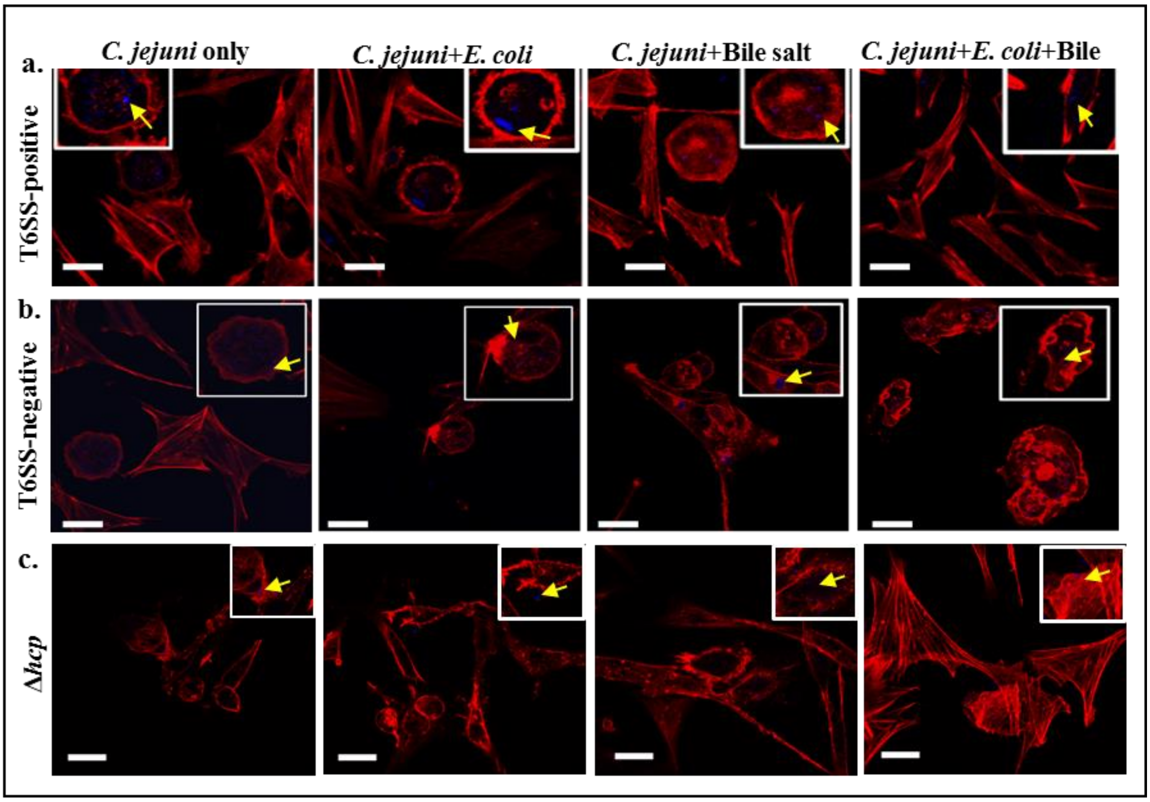

Fig. S8: **(a, b, c)** Representative images of *C. jejuni* (stained with DAPI; blue color) invasion of CEICs (membrane stained with phalloidin, red color) (Scale bar: 50 µm) indicate that in the presence of prey, only a few T6SS-positive *C. jejuni* could be detected (blue color), compared to the cells infected with *C. jejuni* in the absence of prey (a). In both cases, cells were grown in 0.05 % bile salt. Comparative analysis of the images indicates marked changes in cellular architecture characterized by rounding off in the absence of prey (a). Moreover, in the presence or absence of prey or bile salt, the changes in CEICs remained consistent and highly evident when infected with T6SS-negative (b) or Δ*hcp* (c) *C. jejuni*.

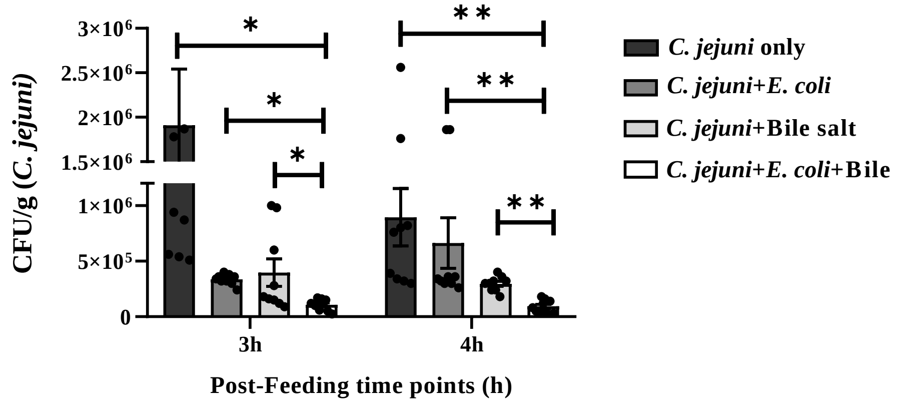

a.

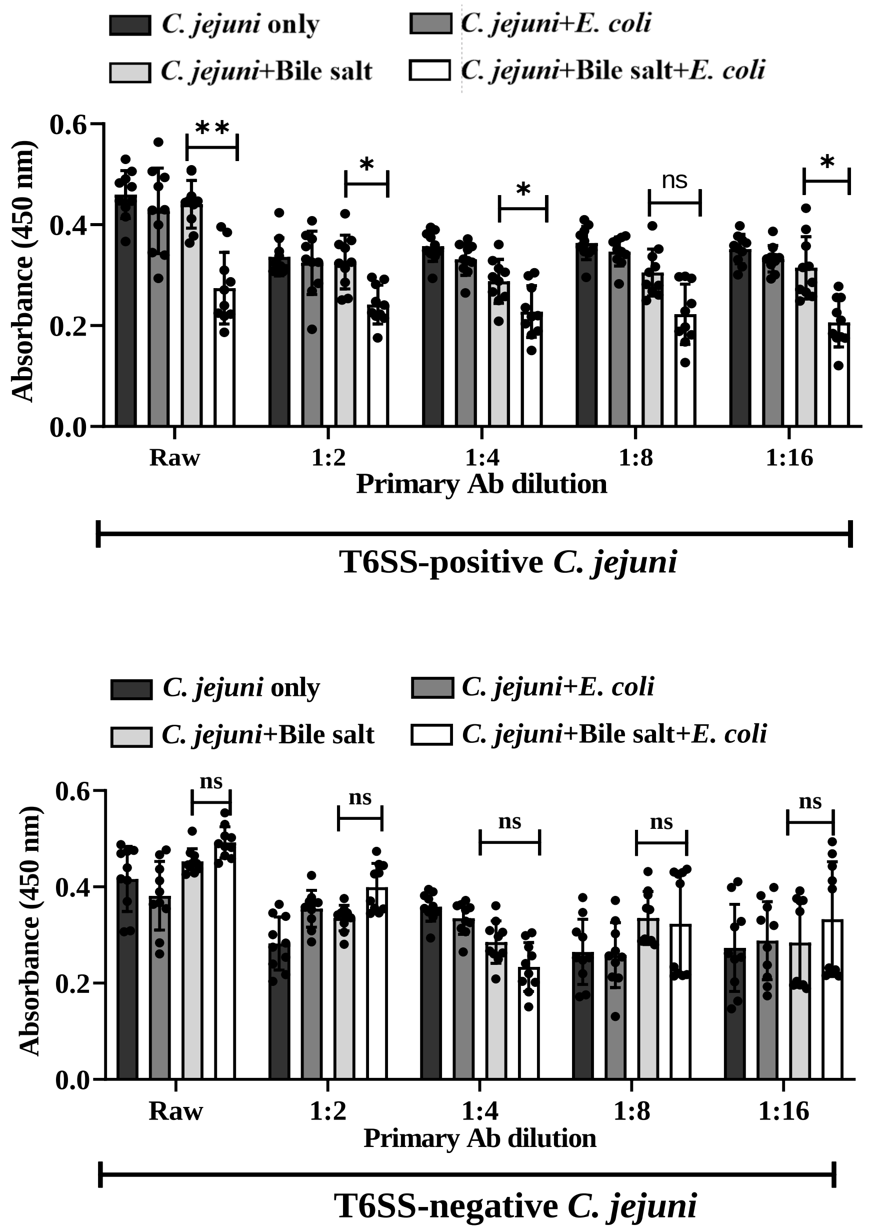

b.

Fig. S9: Prey-dependent depletion of *C. jejuni* and reduction in anti-*C. jejuni* antibody in chickens maintained on bile salt supplementation.

a. *C. jejuni* count in freshly collected fecal samples from experimental chickens at different post-feeding times (3h and 4h). Birds were orally administered with 1×10^7 CFU of *C. jejuni*, 2×10^8 CFU

of *E. coli* and 0.2 % of bile salt in different combinations. Data indicate a significant reduction of *C. jejuni* in fecal pellet when birds received *C. jejuni* and *E. coli* in the presence of bile salt. All error bars represent the mean± standard deviation (mean ± SD) (n = 10).
b. Comparative analysis of anti-*C. jejuni* antibody titer in the chicken intestinal lavage (sIgA). Indirect ELISA was performed using serial dilution (2-fold) of lavage samples. Data show that in the presence of bile salt, the sIgA titer substantially decreased when chickens were administered with T6SS-positive *C. jejuni* and *E. coli*. However, no such changes were observed when T6SS-negative *C. jejuni* was used.

References

1. Jayaraman S, Das PP, Saini PC, Roy B, Chatterjee PN. Use of Bacillus Subtilis PB6 as a potential antibiotic growth promoter replacement in improving performance of broiler birds. Poult Sci. 2017;96:2614–22.

2. Lahiri A, Bhowmick S, Sharif S, Mallick AI. Pre-treatment with chicken IL-17A secreted by bioengineered LAB vector protects chicken embryo fibroblasts against Influenza Type A Virus (IAV) infection. Mol Immunol. 2021;140:106–19.
